# Molecular constraints on CDR3 for thymic selection of MHC-restricted TCRs from a random pre-selection repertoire

**DOI:** 10.1038/s41467-019-08906-7

**Published:** 2019-03-04

**Authors:** Jinghua Lu, François Van Laethem, Abhisek Bhattacharya, Marco Craveiro, Ingrid Saba, Jonathan Chu, Nicholas C. Love, Anastasia Tikhonova, Sergei Radaev, Xiaoping Sun, Annette Ko, Tomer Arnon, Eric Shifrut, Nir Friedman, Nan-Ping Weng, Alfred Singer, Peter D. Sun

**Affiliations:** 1 Structural Immunology Section, Laboratory of Immunogenetics, National Institute of Allergy and Infectious Diseases, Rockville, MD 20852 USA; 2 Experimental Immunology Branch, National Cancer Institute, Bethesda, MD 20892 USA; 3 Lymphocyte Differentiation Section, Laboratory of Molecular Biology and Immunology, National Institute on Aging, National Institute of Health, Baltimore, MD 21224 USA; 4 Department of Immunology, Weizmann Institute of Science, 76100 Rehovot, Israel; 5 Department of Physics and Astronomy, Alfred University, 1 Saxon Drive, Alfred, NY 14802 USA

## Abstract

The αβ T cell receptor (TCR) repertoire on mature T cells is selected in the thymus, but the basis for thymic selection of MHC-restricted TCRs from a randomly generated pre-selection repertoire is not known. Here we perform comparative repertoire sequence analyses of pre-selection and post-selection TCR from multiple MHC-sufficient and MHC-deficient mouse strains, and find that MHC-restricted and MHC-independent TCRs are primarily distinguished by features in their non-germline CDR3 regions, with many pre-selection CDR3 sequences not compatible with MHC-binding. Thymic selection of MHC-independent TCR is largely unconstrained, but the selection of MHC-specific TCR is restricted by both CDR3 length and specific amino acid usage. MHC-restriction disfavors TCR with CDR3 longer than 13 amino acids, limits positively charged and hydrophobic amino acids in CDR3β, and clonally deletes TCRs with cysteines in their CDR3 peptide-binding regions. Together, these MHC-imposed structural constraints form the basis to shape VDJ recombination sequences into MHC-restricted repertoires.

## Abstract (web summary)

For T cells, functional antigen receptors are selected in the thymus from a pre-selection repertoire by interaction with self MHCs. Here the authors show that specific, non-germline coded features located in the complementarity determining region 3 of the pre-selection antigen receptors are essential for the selection of MHC-restricted repertoire.

## Introduction

Immune protection depends on gene recombination events to randomly generate a huge diversity of antigen receptors able to recognize both existing and future pathogens^1^. Unlike other antigen receptors, αβ T cell antigen receptors (TCRs) normally do not recognize conformational antigenic epitopes but instead are selected in the thymus to recognize linear antigenic peptides bound to major histocompatibility complexes (pMHC)^2^, a feature known as MHC-restriction. MHC-restriction is the canonical feature of T cell antigen recognition and is the basis for unique T cell functions such as immune surveillance because MHC-restriction enables T cells to identify and eliminate cells containing viral or mutated proteins. Despite the critical relationship between MHC restriction and T cell function, the molecular basis for thymic selection of MHC-restricted (MHCr) TCRs remains largely unknown.

Immature T cells in the thymus express TCRs, which are then screened for ligand specificity during thymic events known as positive and negative selection^3,4^. Thymocytes bearing TCRs that engage intra-thymic ligands and transduce intra-cellular TCR signals are selected to survive and to undergo further differentiation, whereas thymocytes that are not signaled by their TCR undergo death-by-neglect. TCR signals are transduced by the protein tyrosine kinase Lck which, in wildtype thymocytes, is bound to the cytosolic tails of CD4/CD8 coreceptor proteins away from the TCR. Consequently, TCR signaling of thymic selection in wildtype thymocytes requires TCRs to access coreceptor-bound Lck by binding to the identical pMHC ligands as do the CD4/CD8 coreceptor proteins^5–8^. Because CD4/CD8 coreceptors only bind to MHC molecules, thymic selection in wildtype mice generates only MHCr TCR repertoires that recognize linear antigenic peptides presented by MHC. However, genetic deletion of both CD4 and CD8 coreceptor proteins in developing thymocytes makes Lck available to all TCRs, which can then signal thymic selection upon engagement of any intra-thymic ligand. As a result, thymic selection in mice genetically deficient in both co-receptor proteins and MHC (B2m^−/−^H-2Ab1^−/−^CD4^−/−^CD8a^−/−^ mice, referred to as Quad-deficient mice) generates MHC-independent (MHCi) TCRs that resemble antibodies in recognizing native conformational antigenic epitopes independently of MHC^5–8^.

We undertake the present study to compare TCR repertoire sequences derived from pre-selection developing thymocytes, as well as mature MHCr, and MHCi T cells in an attempt to reveal requirements for thymic selection of MHCr TCRs. We further use mice expressing a pro-survival Bcl-2 transgene in vivo to distinguish mechanistically the removal of TCRs as results of clonal deletion or failed signaling. Here we find that the pre-selection TCR repertoire contains TCR sequences both compatible and in-conflict with MHC binding. Most surprisingly, we find that thymic selection distinguishes MHCr and MHCi TCRs primarily by their TCR-CDR3 segments, which are hyper-variable and non-germline encoded. Thus, this study identifies molecular constraints imposed on hyper-variable TCR-CDR3 segments that are required for thymic selection of MHCr TCR repertoires.

## Results

### Characterization of MHCr and MHCi TCR repertoires

In this study, we considered that peripheral TCR from MHC-deficient Quad^ko^ (Q) animals constitute an MHCi repertoire that is selected in the thymus by interactions with native ligands, whereas TCR from wildtype mice are MHCr and are selected in the thymus by interactions with peptide-MHC complexes^6^. However, it is not clear if these TCR populations form distinct repertoires, or if MHCi TCRs are a subset of MHCr TCRs that fortuitously bind to native ligands in the absence of MHC, as has been suggested^9^. To distinguish these two possibilities, we performed deep RNA sequencing (RNAseq) of individual TCRα and TCRβ chains from MHCi Quad^ko^ (Q) and Quad^ko^Bcl-2^tg^ (QB) mice^5,6,8^ and compared their sequences to TCR from three MHCr wildtype strains (B6, BALB/c, B10.BR) with different MHC haplotypes (H-2^b^, H-2^d^, H-2^k^) (Supplementary Table 1-3).

**Fig. 1:**
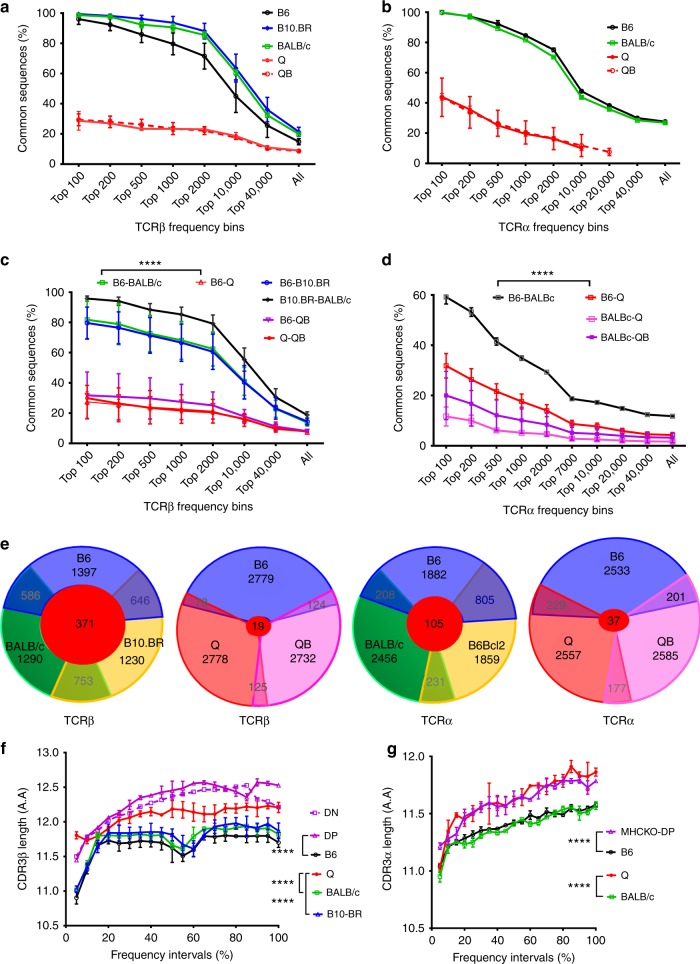
Overall αβTCR sequence overlap comparisons among various MHCr and MHCi repertoires. **a**, **b** Percentage of TCRβ (**a**) and TCRα (**b**) sequence overlaps observed within each strain of B6, B10.BR, BALB/c, Quad^ko^ (Q), or Quad^ko^Bcl-2^tg^ (QB) animals. **c**, **d** Percentage of TCRβ (**c**) and TCRα (**d**) sequence overlaps observed between different strains of animals. The pair-wise sequence overlapping percentages are calculated using top 100, 200, 500, 1000, 2000, 10,000, 40,000 highest frequency and all sequences of each strain, respectively. **e** TCRβ and TCRα common sequences overlapping among the top 3000 sequences from B6, B10.BR, BALB/c, Quad^ko^ (Q), or Quad^ko^Bcl-2^tg^ (QB). **f**, **g** The distribution of average length of CDR3β (**f**) and CDR3α (**g**) in MHCr and MHCi TCR repertoires with respect to their frequency intervals. Sequences from each strain were grouped by 5% intervals from the top 5% frequent sequences until the accumulation of all sequences (100%). The error bars indicate standard deviations from the means. The p-values all figures are calculated using unpaired student *t-*test with * = *p* ≤ 0.05, ** = *p* ≤ 0.01, *** ≤ 0.001, **** = *p* ≤ 0.0001. The results included in all panels are from at least two independent experiments with all data included

As an initial characterization of these repertoires, we assessed the presence of ‘common sequences’, which are identical TCR amino acid sequences, shared within the same mouse strain. In wildtype B6 mice with MHCr repertoires, ~20% of all TCRα/β amino acid sequences were found common to more than one B6 mouse (Fig. 1a, b). Interestingly, TCR sequences that were present in high frequency in one B6 mouse were often detected in other B6 mice, with the repertoire overlap between B6 mice approaching 100% for the most frequent (i.e. top 100) TCR sequences (Fig. 1a, b)^10,11^. Similarly, high repertoire overlaps were also found between individual BALB/c mice and individual B10.BR mice, and their within strain overlaps also approached 100% for the most frequent (top 100) TCR sequences (Fig. 1a, b). In contrast, Quad^ko^ and Quad^ko^Bcl-2^tg^ mice with MHCi repertoires had significantly lower repertoire overlaps (<40%) between individual mice for the most frequent (top 100) TCR sequences.

We also asked if common sequences were shared between mice of different strains. In fact, TCRα/β sequences from MHCr strains exhibited significant commonality between different MHCr strains, as overlap between MHCr strains exceeded 80% for the most frequent (top 100) sequences (Fig. 1c, d), so that TCR amino acid sequences that were present in high frequency in one MHCr mouse strain were likely also present in another MHCr mouse strain (Fig. 1c, d). However, such commonality between MHCr stains did not extend to MHCi strains, as sequence overlap between B6 and Quad^ko^ or between B6 and Quad^ko^Bcl-2^tg^ mice only approached 30% for most frequent (top 100) sequences (Fig. 1c, d). Thus, MHCr repertoires exhibit significantly higher sequence conservation than MHCi repertoires, whose lack of shared sequences resemble those of antibody repertoires^12^.

To further illustrate differences in sequence overlap between MHCr and MHCi repertoires, we constructed Venn diagrams for the 3000 most frequent TCRβ and TCRα sequences among individual animals from three different strains (Fig. 1e). For three MHCr animals, one third of the 3000 TCRβ sequences are shared between individual animals (e.g. 1017 between B6 and B10.BR), with 371 sequences common to all three MHCr strains. However, few of the top 3000 TCRβ sequences in the B6 mouse (~100 out of 3000) were shared with either Quad^ko^ or Quad^ko^Bcl-2^tg^ mice, with only 19 TCRβ sequences common to B6 and both MHCi repertoires (Fig. 1e). Thus, MHCr TCRβ and TCRα sequences are more conserved than those of MHCi sequences.

### TCR-CDR3 have distinct features in MHCr and MHCi repertoires

**Fig. 2:**
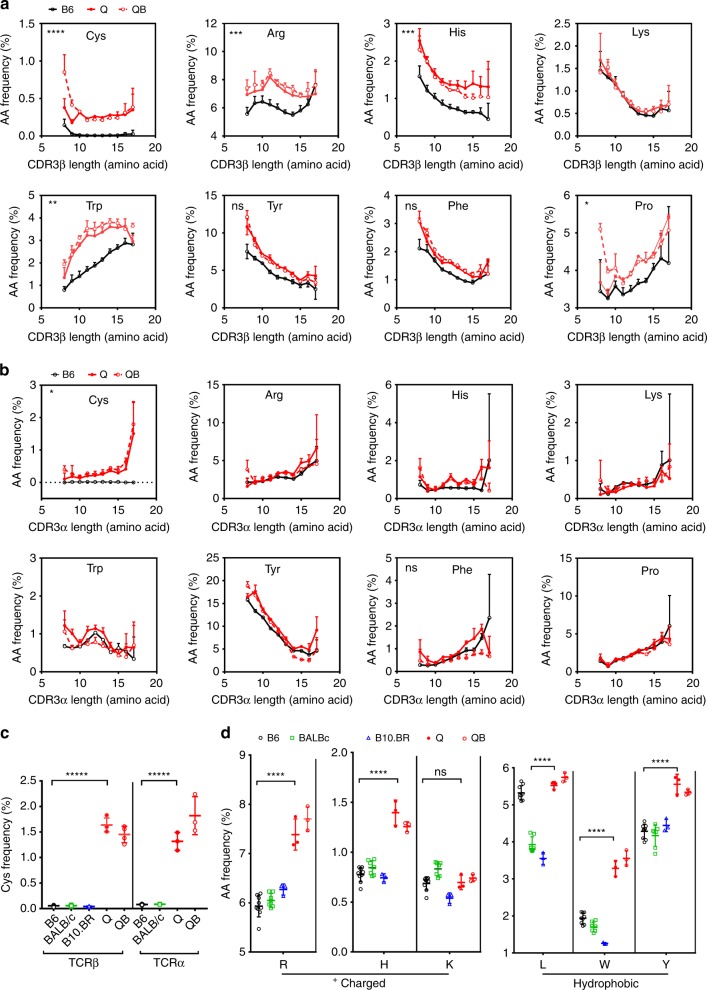
CDR3 length dependent amino acid usage. **a**, **b** The frequencies of amino acids in FG-loops of CDR3β (**a**) and CDR3α (**b**) in B6, Q and QB repertoires with respect to their CDR3 length. **c** Cys usage of CDR3β (left panel) and CDR3α (right panel) is significantly lower in MHCr repertoires than MHCi repertoires. Our analysis excludes a germline-encoded CDR3 cysteine present in several mouse Vβ gene families because that cysteine is not in the FGβ-loop and does not contact peptide-MHC. **d** Usage of charged and hydrophobic amino acid usages of MHCr repertoires and MHCi repertoires

To further compare MHCr and MHCi repertoires, we examined their non-germline-encoded hyper-variable TCR-CDR3 segments. Regarding CDR3 length distributions, we found that CDR3β and CDR3α segments in MHCr repertoires (from B6, BALB/c, and B10.BR mice) were significantly shorter than in MHCi repertoires (from Q and QB mice) and this was the case throughout the entire TCR frequency range (Fig. 1f, g and Supplementary Fig 1), indicating that shorter TCR-CDR3 segments was a specific feature of MHCr TCRs. We then assessed TCR-CDR3 amino acid usage and specifically focused on amino acids in the peptide and MHC-contact region of CDR3, which is structurally known as the FG-loop in IgV domain and consists of non-germline-encoded amino acids in positions 108–114 (Supplementary Fig. 2a–b)^13,14^. We calculated the usage frequency of each amino acid in the FG-loops of both TCRβ and TCRα chains from B6, Q, and QB mice (Fig. 2a, b and Supplementary Fig 2c, d). Gly, Ala, Asp, Asn are the most abundant amino acids in both FGβ- and FGα-loops, and their usages do not differ between B6 and the MHCi mice (Supplementary Fig 2c–d). In contrast, Cys is only present in the FGβ- and FGα-loops of Q and QB but is virtually absent from B6 CDR3β/α (Fig. 2a, b). In fact, Cys is absent from the FGβ- and FGα-loops of all MHCr strains (B6, B10.BR, and BALB/c) (Fig. 2c) as well as from their individual Vβ and Vα gene families (Supplementary Fig. 2e–f). Thus, Cys is specifically excluded from the FG-loops of MHCr repertoires but not MHCi repertoires. Less dramatic than differences in Cys usage, FGβ-loop usages of positively charged amino acids (Arg and His) and hydrophobic amino acids (Trp, Tyr and Pro) are significantly reduced in MHCr TCRs (B6, B10.BR and BALB/c) compared to MHCi TCRs (Q and QB) (Fig. 2a, d); notably, FGα-loop usages of these amino acids are not reduced (Fig. 2b). We conclude that, compared to MHCi repertoires, MHCr repertoires have shorter CDR3 segments, lack Cys in both CDR3β and CDR3α peptide-contacting regions, and have reduced FGβ-loop usage of positively charged and hydrophobic amino acids.

### High sequence diversity of pre-selection repertoires

**Fig. 3:**
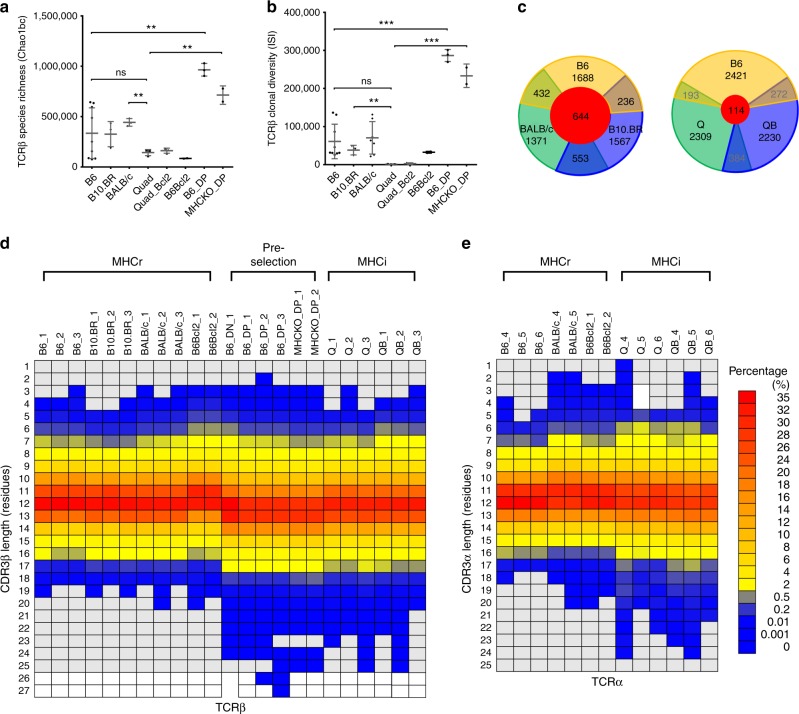
Sequence diversity and CDR3 length distributions. **a** TCRβ species richness of various TCRβ repertoires based on Chaobc1 analysis. **b** The inverse simpson index (ISI) was calculated to estimate the clonal diversity of various TCRβ repertoires. **c** TCRβ sequence overlapping among the top 3000 common sequences to B6 DP1 from B6, B10.BR, BALB/c, Quad^ko^ (Q), or Quad^ko^Bcl-2^tg^ (QB). **d**, **e** Heatmap showing CDR3 length distributions of TCRβ (**d**) and TCRα (**e**) for various MHCr, MHCi and pre-selection repertoires. The mice are identified with a number associated with their strain name (Table S1-S2). For strains with more than three sequencing attempts, only three repertoires are shown here

To determine a possible basis for these repertoire differences, we considered that they might be due to differences in the thymic selection requirements of MHCr and MHCi TCRs. To explore this possibility, we sequenced pre-selection TCRα/β chains from unsignaled CD69^−^CD4^−^CD8^−^ (double negative, DN) thymocytes and unsignaled CD69^−^CD4^+^CD8^+^ (double positive, DP) thymocytes from B6 and MHC-deficient (*H2Ab1*^−/−^*B2m*^−/−^) mice^15^ (Supplementary Table 1–3). Interestingly, TCRβ repertoires from pre-selection DP thymocytes are significantly more diverse than those from mature MHCr and MHCi T cells as measured by both Chao1 and Inverse Simpson Index (ISI)(Fig. 3a, b)^16,17^. This is consistent with the pre-selection repertoire including both MHCr and MHCi TCR sequences. To illustrate this further, we identified the top 3000 sequences that were identical between pre-selection DP TCRs and mature B6 TCRs. Approximately 50% of the pre-selection sequences that were shared with B6 were also found in BALB/c and B10.BR mature TCR repertoires (Fig. 3c), but far fewer (<20%) were found in Q or QB repertoires. Thus, the pre-selection sequences that the thymus selects into MHCr TCR repertoires are mostly non-overlapping with those leading to MHCi TCR repertoires.

### Thymic selection limits the CDR3 length of MHCr TCRs

We then considered that shorter CDR3 amino acid length distributions displayed by MHCr TCRs compared to MHCi TCRs might be due to different requirements for thymic selection of MHCr and MHCi repertoires. We found that CDR3 amino acid length distributions of pre-selection TCR chains in DN and DP thymocytes that have not undergone thymic selection were significantly longer than those in post-selection mature MHCr TCRs and resembled those of post-selection MHCi TCRs throughout the entire TCR frequency range (Fig. 1f, g, Supplementary Fig. 3). To assess the effect of thymic selection on CDR3 length further, we constructed a heatmap of CDR3 length distributions of MHCr, MHCi, and pre-selection TCRs from multiple mice (Fig. 3d, e). The CDR3 length heatmap showed that CDR3β and CDR3α segments in all MHCr animals contained 2–18 amino acids (aa) with none exceeding 20aa (Fig. 3d, e). In contrast, the CDR3 length of pre-selection TCR chains extended beyond 20aa, albeit in relatively low frequencies as was also the case for mature MHCi TCRs (Fig. 3d, e). The absence of longer CDR3 segments from MHCr repertoires might either be because longer CDR3 segments signaled MHC-specific clonal deletion in the thymus or because they failed to generate any MHC-specific signals and underwent ‘death-by-neglect’. To distinguish these two possibilities, we assessed if TCRs with longer CDR3 segments would be detected in B6Bcl-2^tg^ mice, which express a pro-survival Bcl-2^tg^ that prevents clonal deletion but does not prevent death-by-neglect^4^. In fact, CDR3 segments >20aa long were not rescued in B6Bcl-2^Tg^ mice (Fig. 3d, e), indicating that such TCRs underwent death-by-neglect, not clonal deletion, during MHC-specific selection.

**Fig. 4:**
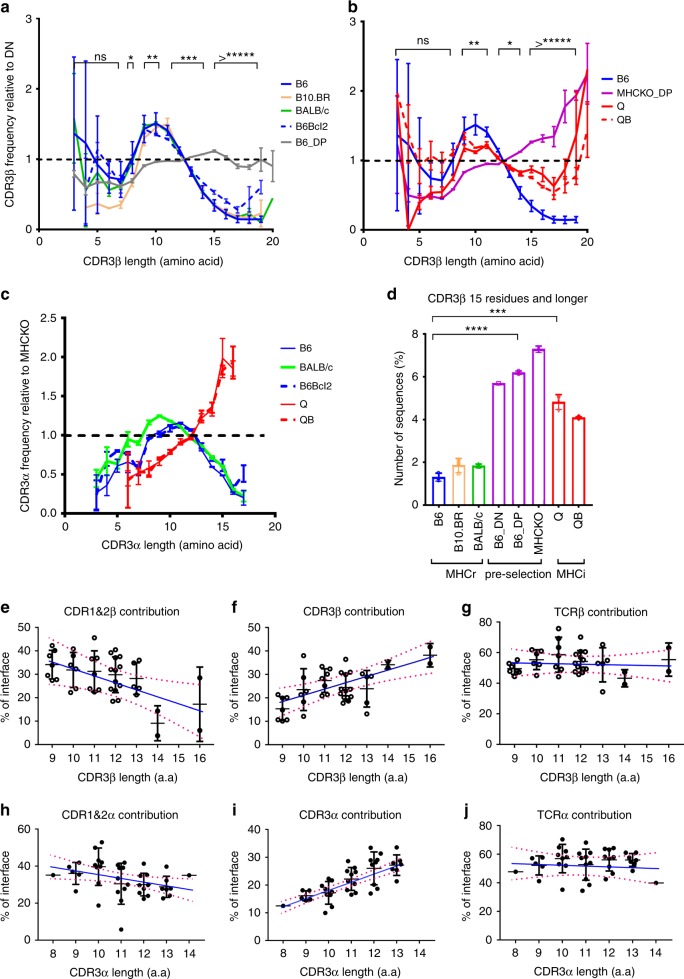
Normalized CDR3 length distributions. **a**–**c** Comparisons of normalized CDR3 length distributions of TCRβ (**a–b**) and TCRα (**c**) among MHCr, MHCi and pre-selection repertoires. The repertoire-specific preference of CDR3 length distributions is indicated by respective fold changes when normalized to B6 DN for TCRβ (**a, b**) or the average of MHCKO DP for TCRα (**c**) sequences. Unless otherwise noted, the CDR3 length and all other analyses reported here were carried out using all repertoire data listed in Supplementary Tables 1 & 2 without rejection and regardless of the sequencing depth variation. **d** MHCr repertoires contain fewer TCRβ sequences with CDR3 length of 15 or longer than MHCi and pre-selection repertoires. **e**–**j** The MHC contact area from CDR3 and germline CDR1&2 displayed as percentage of total TCR-MHC interface area in each MHC-TCR complex structures from Protein Data Bank(PDB). Panels **e**–**j** are fitted with linear regression using GraphPad Prism with dotted lines indicating 95% confidence

We conclude that short CDR3 amino acid length distributions in MHCr TCRs is not an intrinsic feature of TCR structure (since pre-selection and MHCi TCRs display longer CDR3 segments), but rather is a feature of MHCr TCR that is imposed during thymic selection. To further assess thymic influence on CDR3 length, we normalized the CDR3 length distributions of each repertoire to that of pre-selection DN thymocytes (Fig. 4a–d). Such normalizations revealed that mature B6 repertoires favor shorter CDR3 of  8–13 aa and disfavor longer CDR3 compared to pre-selection repertoires, whereas MHCi repertoires do not exhibit such bias in CDR3. As a result, MHCr repertoires contained 3-fold fewer TCRs with CDR3β segments >15aa long than pre-selection or MHCi repertoires (*p* < 0.001) (Fig. 4d). The preference in MHCr repertoires for shorter CDR3 sequences indeed occurs in individual V-genes, as shown here in TRBV1 for one representative example (Supplementary Fig. 4a). These data are consistent with structural data in the Protein Data Bank (PDB) which shows a majority of MHCr CDR3α and CDR3β segments of 8–13 aa long (Supplementary Fig. 4b).

To understand why shorter CDR3 segments were favored by MHCr TCRs, we analyzed 42 αβTCR-MHC structures in the protein data bank (PDB) to assess the impact, if any, of longer CDR3 on MHC-binding (Fig. 4e–j). The results showed that as CDR3α and CDR3β lengths increased, their contribution to TCR-MHC interface area also increased (Fig. 4f, i), but the contact between TCR-CDR1/2 and MHC decreased (Fig. 4e, h). Based on these sequence and structural analyses, we conclude that CDR3 lengths of 8–13 aa long are optimal for promoting MHC-binding because longer CDR3 reduce CDR1/2 contacts with MHC and, and as a result, impair TCR binding to MHC.

### MHCr CDR3 FG-loops exhibit similar amino acid usage

Next, we analyzed the impact of thymic selection on CDR3 amino acid usage. To do so, we normalized FGβ- and FGα-loop amino acid frequencies to those in pre-selection TCRs and displayed normalized frequencies in a heatmap (Supplementary Fig 5a–b). FG-loop amino acid usage was highly consistent within strains of both MHCr and MHCi repertoires. However, there are notable differences between pre-selection repertoires and the mature MHCr or MHCi TCR repertoires for FG-loop amino acids. Similar differences were also observed in CDR3β segment encoded within their D-gene region (positions 109–112) (Supplementary Fig. 5c), excluding the possibility that these differences were due to V-gene sequencing primer-bias.

**Fig. 5:**
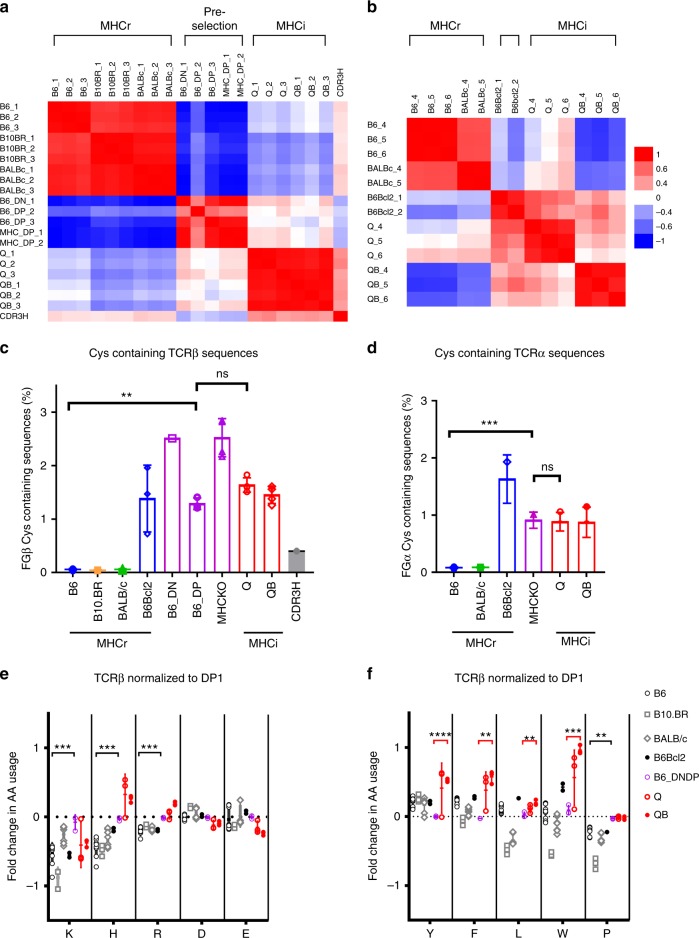
Comparison of Amino acid usage in TCR CDR3 FG-loop from MHCr, MHCi and pre-selection repertoires. **a**, **b** Pearson correlations calculated using normalized amino acid usage among MHCi, MHCr and pre-selection TCR repertoires. The FGβ- (**a**) and FGα-loop (**b**) amino acid usage frequencies of individual repertoires are normalized against those of B6 DP_1 and MHCKO DP, respectively. The pair-wise Pearson correlation coefficients were displayed as heatmaps to indicate the distinct amino acid usages of MHCi, MHCr and pre-selection TCR repertoires. **c**, **d** The frequency of Cys-containing TCRβ (**c**) and TCRα (**d**) sequences in MHCr, MHCi and the pre-selection repertoires. **e**, **f** Fold changes of charged and hydrophobic amino acid usages observed in various repertoires. Amino acid frequencies are normalized to those in B6_DP1 repertoire

To assess similarities in amino acid usage among various TCRβ repertoires, we calculated Pearson correlation coefficients using normalized amino acid frequencies between individual pairs of animals (Fig. 5a, b). Strikingly, greater amino acid usage similarities are observed within all MHCr, all pre-selection or all MHCi repertoires. That is, mature B6 TCRβ exhibited closer amino acid usage similarity to B10.BR and BALB/c than to immature pre-selection DN and DP sequences. In addition, the amino acid compositions of pre-selection sequences more closely resemble those of mature MHCi repertoires than MHCr repertoires, indicating that thymic selection has a greater impact on CDR3 amino acid usage in MHCr than MHCi repertoires. Nevertheless, CDR3 amino acid usage is not identical between pre-selection and MHCi repertoires, indicating that thymic selection also affects CDR3 amino acid usage in MHCi repertoires.

### Clonal deletion eliminates cysteines from MHCr FG-loop

Cysteines were present in both FGβ- and FGα-loops of pre-selection TCR at frequencies of ~1–2%, which reflects its D-gene coding frequency (Supplementary Fig 5d–e). Cysteines were present at similar frequencies in MHCi TCRs, but were nearly absent from FG-loops and Dβ-regions of MHCr TCRs (Fig. 5c, d, Supplementary Fig 5f). In absolute terms, there are 5000–15000 FGβ-loop cysteine-containing TCRs in pre-selection sequences but less than 200 in mature MHCr sequences in mature repertoires of 10^5^–10^6^ sequences. Notably, the exclusion of cysteines from TCR FG-loops in MHCr repertoires was unique to cysteine, as methionine was not excluded even though both are sulfur-containing amino acids and both are expressed at similar frequencies in pre-selection and MHCi repertoires (Supplementary Fig. 5g–h). To determine if TCRs with FG-loop cysteines signaled clonal deletion during MHC-dependent selection, we assessed FG-loop cysteine usage in B6Bcl-2 mice expressing the pro-survival Bcl-2^tg^ that restores TCRs eliminated by clonal deletion^4^. We found that the Bcl-2^tg^ completely restored the appearance of FG-loop cysteine-containing TCRs (Fig. 5c, d, Supplementary Fig 5f). These results reveal that FG-loop cysteines are absent from MHCr sequences because they induce clonal deletion during thymic selection.

As a possible explanation, we suggest that FG-loop cysteines form disulfide-bonds with free cysteines in MHC-bound peptides which are present in the thymus^18^, leading to clonal deletion during MHC-specific thymic selection. In contrast FG-loop cysteines do not affect MHC-independent thymic selection because MHC-independent ligands are extra-cellular proteins that very rarely contain free cysteines that could link to free cysteines in MHCi TCR.

### Positively charged residues are restricted in MHCr FGβ-loop

When normalized against pre-selection sequences, the FGβ-loop usage of positively charged Lys, His, and Arg as well as proline were selectively reduced in MHCr (*p* < 0.005), but not in MHCi repertoires (except Lys) during thymic selection (Fig. 5e, f). Because expression of the Bcl-2^tg^ did not increase the frequencies of these four amino acids (K,H,R, and P) in MHCr FGβ-loop in B6Bcl-2^tg^ mice (Fig. 5e), we conclude that sequences with excessive number of positive charged amino acids and proline in their FGβ-loops did not induce clonal deletion but, instead, impaired MHC-specific TCR signaling so that their thymocytes underwent death by neglect.

To understand why positively charged amino acids might specifically impair MHC-dependent TCR signaling, we analyzed TCR contact regions on murine MHC molecules in the PDB databank (www.rcsb.org) (Supplementary Fig 5i–j). Interestingly, the CDR3β contact regions on the α2-helices of class I MHC H-2K and H-2D, and the CDR3β contact regions on the β1-helices of class II MHC H2-Aβ and H2-Eβ all contain positively charged Lys and Arg residues, as evident from their surface electrostatic potential distributions (Supplementary Fig. 5k). Specifically, a cluster of three positively charged residues (Arg^144^–Arg^145^–Lys^146^ or Lys^144^–His^145^–Lys^146^) at the CDR3β contact region is conserved in mouse and human class I MHC molecules (Supplementary Fig. 5l). Regarding MHC class II, two conserved positively charged residues are present in the CDR3β contact region of MHC-II β1-helix (Supplementary Fig 5j, 5l). Consistent with more positively charged residues in the CDR3β contact region of MHC class I than MHC class II molecules, positively charged amino acid usage was more discriminated in MHC class I-specific CD8 TCR than in MHC class II-specific CD4 TCR (Supplementary Fig. 5m). Thus, positively charged FGβ-loop residues interact unfavorably with positively charged residues on MHC to impair MHC-specific TCR binding and signaling. In contrast, the CDR3α contact regions on both class I and class II MHC molecules are less charged (Supplementary Fig. 5i–k), resulting in no restriction to charged amino acids in TCR FGα-loop sequences.

Regarding FGβ-loop usage of proline, a structurally rigid amino acid, we suggest that FGβ-loop prolines may impair MHC-specific selection by hindering CDR3β loops from adopting conformations optimal for MHC binding.

### Thymic selection affects usage of hydrophobic amino acids

Our analysis also revealed that, compared to pre-selection repertoires, FGβ-loop usages of the four hydrophobic amino acids (Y,F,L, and W) were increased in MHCi repertoires (Supplementary Fig. 5a–b), suggesting they are favored for MHCi selection. In contrast, we observed reduced FGβ-loop usage of Leu and Trp (L, and W), in some MHC-restricted repertoires, indicating that usage of these hydrophobic amino acids is less favored by MHC-specific than MHCi thymic selection (Fig. 5f). Indeed, the higher frequencies of Trp and Tyr in MHCi TCRs are similar to those in antibody CDR3H and are significantly higher than their overall frequencies in mammalian proteins (Supplementary Fig. 5n)^19^. The frequent occurrence of Trp and Tyr in antibody CDR3H presumably reflects hydrophobic interactions that enhance ligand binding affinity. Consequently, we suggest that FG-loop hydrophobic amino acids may enhance non-specific TCR-ligand binding, leading to increases in MHCi selection and MHCr clonal deletion. Consistently, FG-loop usage of Leu and Trp (L,W) has been associated with autoreactivity in MHC-sufficient mice^20^.

### Effect of thymic selection on V- and J-gene usages

**Fig. 6:**
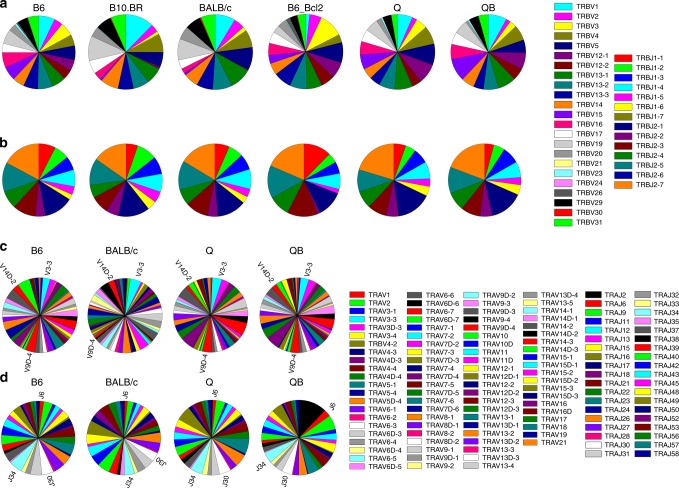
V-gene and J-gene usage in various repertoires. **a**, **b** Representative Vβ gene (**a**) and Jβ gene (**b**) usage of MHCr, MHCi and pre-selection repertoires. **c**, **d** Representative Vα gene (**c**) and Jα gene (**d**) usage of MHCr and pre-selection repertoires

**Fig. 7:**
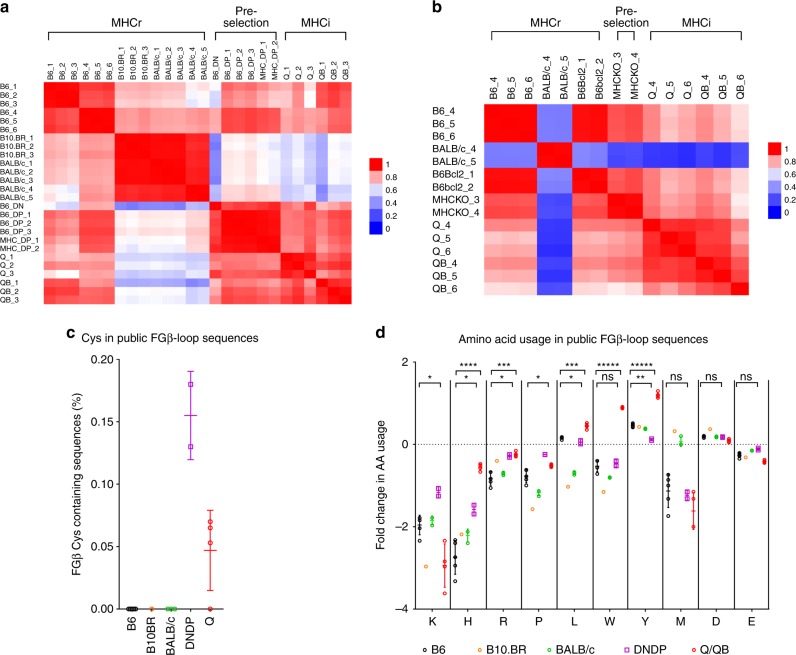
V-gene usage comparison and the usage of common sequences. **a**, **b** Pair-wise Pearson correlation coefficients were calculated using Vβ-gene (**a**) and Vα-gene (**b**) frequencies and displayed as heatmaps for MHCi, MHCr and pre-selection TCR repertoires. The sequences of Vβ- and Vα-genes were from Adaptive Biotechnology and Irepertoire, respectively. **c**, **d** Cys and other amino acid usages of CDR3β FG-loop among public sequences from MHCr or MHCi repertoires. The fold change is calculated with respect to those present in B6_DN repertoire. The public sequences of Q and QB are analyzed together

The influence of thymic selection on TCR CDR3 region prompted us to examine if thymic selection also affects the usage of germline-encoded V- and J-genes, as well as their pairings (Fig. 6, Supplementary Fig 6). Overall, the observed frequencies of germline-encoded Vα-,Vβ-, Jα-, and Jβ-genes were quite similar among pre-selection, MHCr and MHCi repertoires with sequences from animals of the same strain exhibiting highest similarities (Fig. 6a–d, Fig. 7a, b, Supplementary Fig. 6a)^21^. While the usage variations of these germline-encoded segments were also present (Fig. 7a, b), there were no significant preferences separating MHCr from MHCi sequences. In fact, the Vβ and Vα usages of B6 resemble those of Q and QB more than those of B10.BR and BALB/c (Fig. 7a, b). Likewise, the Jβ usages of B6 resemble those of Q and QB more than those of pre-selection sequences (Supplementary Fig 6a), suggesting that the majority of V- and J-gene frequencies were not affected by MHC-dependent thymic selection.

Similarly, while VJ pairing occurred in near combinatorial fashion (Supplementary Fig. 6b–f), the VJ pairing frequency distributions of B6 correlated more to those of MHCi animals than to MHCr BALB/c (Supplementary Fig. 6g–h). Thus, neither V- and J-gene usages nor their pairing are significantly affected by thymic selection.

### MHC-restriction is reflected in public TCR sequences

Public TCR sequences are identical sequences found in at least three animals. Consequently, we thought it reasonable that the molecular characteristics of MHC-restriction would be exemplified in public TCR sequences in MHCr mice. Indeed, public sequences showcase the effect of MHC-imposed constraints on FGβ-loop sequences (Fig. 7c, d). That is, relative to the pre-selection repertoires, Cys was absent and the usage of positively charged K,H,R and P were markedly reduced, while negatively charged D and E were unaffected in their FGβ-loops. Thus, public sequences bear the hallmark features of MHC-restriction. As the peptide specificities are expected to vary among B6, B10.BR and BALB/c, these conserved amino acid usages likely reflect constraints related to MHC rather than individual peptide specificities.

### MHC-restriction improves peripheral fitness of TCR

**Fig. 8:**
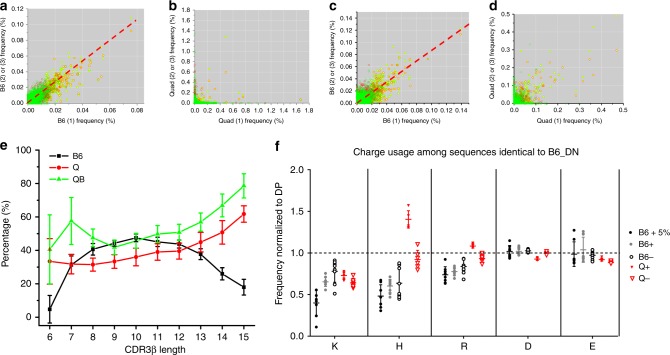
Frequency scatter plots. **a**, **c** Frequency scatter plots among MHC-restricted TCRβ (**a**) and TCRα (**c**) repertoires from individual B6 mice. **b**, **d** Frequency scatter plots among MHCi TCRβ (**b**) and TCRα (**d**) repertoires from individual Quad^ko^ sequences. Each panel contains the comparison of three independent sequence repertoires from each strain of mice with the horizontal axis representing the frequencies of the first repertoire sequences, vertical axis representing the frequencies of the second (red circle) and third (green circle) repertoires. Common sequences between repertoire 1 and 2 are shown as red circles in the first quadrant with horizontal and vertical coordinates representing their respective frequencies in repertoire 1 and 2. Common sequences between repertoire 1 and 3 are shown as green circles with horizontal and vertical coordinates representing their respective frequencies in repertoire 1 and 3. Non-overlapping sequences appear on their respective axes. Top 200 common sequences present in all three repertoires are shown as filled yellow circles. Linear regressions of the frequency distributions between B6 repertoires 1 and 2 are shown in red lines. **e** CDR3β length-dependent expansion of MHCr and MHCi sequences from pre-selection repertoires. The percentage of expanded TCRβ sequences during thymic selection were plotted according to their CDR3 length for B6, Quad^ko^ and Quad^ko^Bcl-2^tg^. The analysis included common sequences between pre-selection (B6_DN) and MHCr (B6) or MHCi (Q and QB mice) repertoires. Expansion is defined as sequences whose mature frequencies (normalized for all comparing repertoires) were higher than their pre-selection ones. **f** Charged amino acid usages of CDR3β FG-loop in common sequences between pre-selection and MHCr or MHCi repertoires. B6 (+ 5%), top 5% TCRβ sequences of B6 repertoires; B6^+^, B6^−^, Q^+^, and Q^−^ represent mature sequences either expanded (+) or not expanded (−) in frequency compared to their pre-selection repertoire

Dominant TCR clonotypes in the periphery (high frequency TCR) not only exhibit better sequence conservation in MHCr than MHCi repertoires (Fig. 1), their frequencies are also more correlated in MHCr (Fig. 8a–d). That is, the high frequency sequences in one B6 mouse are also found in high frequency in other B6 mice, resulting in a near diagonal distribution in frequency scatter plots (Fig. 8a, c). Similarly, dominant clonotypes of BALB/c and B10.BR also exhibited frequency correlations between individual mice (Supplementary Fig 7a–c). In contrast, major TCR clonotypes in MHCi repertoires are less conserved and less correlated in their frequencies (Fig. 8b, d). Following differentiation in the thymus, T cells emigrate to the periphery where their survival requires TCR signaling stimulated by peripheral ligand engagement. TCRs that best engage peripheral ligands generate greater signals and preferentially expand. As a result, TCR sequences expanded in mature repertoires are ‘peripherally fit’^22,23^. As high frequency sequences in MHCr animals bear hallmarks of MHC-restriction, their high degree of frequency correlation suggest that their expansion is driven by common MHC-ligand. In contrast, MHCi sequences lack correlated frequencies, suggesting they recognize different ligands in individual animals. To further assess the influence of MHC-restriction to peripheral fitness of TCR, we selected all TCR sequences that are present in both pre-selection and mature repertoires and examined their peripheral fitness. Sequences exhibiting higher frequency in mature than pre-selection repertoires are deemed peripherally fit. Interestingly, the percentage of peripherally fit sequences in B6 mice varied with CDR3 length and was greatest for TCRs with CDR3β of 8–13aa long, and progressively declined with increasing CDR3β lengths (Fig. 8e). In contrast, the peripherally fit Quad^ko^ and Quad^ko^Bcl-2^tg^ sequences increased with increasing CDR3β lengths (Fig. 8e). Thus, longer CDR3β segments diminished the peripheral fitness of MHCr TCR but increased the peripheral fitness of MHCi TCRs. Like CDR3 length, the usage of positively charged K,H, and R was constrained most among the 5% highest frequency TCR sequences. These results showed that the same constraints important for thymic selection of MHCr TCR sequences also impact their peripheral fitness (Fig. 8f).

## Discussion

This study identifies molecular constraints that are primarily imposed on hyper-variable TCR-CDR3 segments during thymic selection of MHC-restricted TCR repertoires. We show that pre-selection TCR repertoires in DN and DP thymocytes contain receptors capable of MHC-binding but also contain receptors structurally incapable of MHC-binding which can be selected into unconventional MHCi repertoires. Consequently, we conclude that the thymus selects MHCr TCRs from a broader pre-selection repertoire by MHC-imposed constraints primarily on the length and amino acid composition of CDR3 segments.

Functional αβTCR are first assembled on DP thymocytes into pre-selection TCRs, which signal positive and negative selection upon binding to thymic ligands. The conserved structure of pMHC complexes restricts the length of TCR-CDR3 to favor shorter CDR3 of 8–13 amino acids. Structurally, TCRs with CDR3 longer than 13 residues impaired their CDR1 and CDR2 contact with MHC. This is primarily due to the location of CDR3α and CDR3β in the center of TCR-MHC contact interface and thus require the movement of exterior CDR1 and CDR2 segments to accommodate longer CDR3 loops^13,24,25^. Because prevention of clonal deletion in thymus did not rescue TCRs with longer CDR3 segments, we conclude that longer CDR3 impair MHC-binding and TCR signaling of MHC-specific positive selection in the thymus.

MHC-specific positive selection constrained not only TCR-CDR3 length but also its usage of specific amino acids. Most prominently, MHC-specific selection disfavored the inclusion of positively charged amino acids (Lys, His, and Arg) in CDR3 FGβ-loops. This constraint of positively charged amino acids in TCRβ subunit is likely due to the presence of conserved positively charged amino acids near CDR3β contact sites on both MHC class I and class II molecules. Thus, electrostatic repulsion inhibits MHC-binding by TCRs with positively charged amino acids in their CDR3 FGβ-loops. Interestingly, rare TCR with longer CDR3α (15 residues) and multiple positively charged Arg and a proline residues (RPRR) in CDR3β have been found to bind to MHC in a flipped orientation^26^. It is interesting to speculate that electrostatic repulsion between this highly positively charged CDR3 FGβ-loop and positive charges on MHC prevented the TCR to adopt the canonical binding mode, instead bound to MHC in a flipped, orientation^27^. It is worth noting that, first, the MHCr and MHCi usage differences of several amino acids, including Trp, Tyr, Cys, Pro, and Arg, exhibited CDR3β length dependence. In most cases, except Cys, their usage differences remained relative constant for CDR3β of 8–14 residues, but became less significant for CDR3β longer than 15 or shorter than 8 amino acids. These CDR3 length dependent amino acid usage variations are consistent with our current finding that MHC-dependent thymic selection prefers CDR3 lengths of 8–13 amino acids. Thus, MHC-imposed restrictions on CDR3 amino acid usage is compounded by MHC-imposed CDR3 length restrictions. Within the optimal CDR3 length, the amino acid usage differences reflect peptide-MHC binding preferences of TCR which becomes a molecular signature of MHC-restriction. Longer or shorter CDR3 are less optimal for MHC-binding, and progressively lose the molecular signature of MHC-restriction. Second, while MHCr and MHCi CDR3 exhibit biased amino acid compositions, no discernable sequence motifs were found conserved in MHCr repertoire. Namely, MHC-binding restricts the type of amino acids present in TCR CDR3 but does not promote specific sequence motifs, leaving sufficient randomness in amino acid sequences to generate specificities required for binding to individual peptide-MHC ligand.

Clonal deletion also shapes mature MHCr TCR repertoire by eliminating TCRs with excessive affinity for intra-thymic MHC-peptide ligands. The most dramatic impact on CDR3 amino acid usage was the clonal deletion of TCRs containing FG-loop cysteines during MHC-specific thymic selection. Cysteines are present at ~1–3% of TCR in pre-selection and MHCi repertoires^28^, but are absent in all mature MHCr repertories, except in mice expressing the Bcl-2 transgene that prevent clonal deletion. Consequently, we think that MHC-specific TCRs with FG-loop cysteines become crosslinked to cysteine-containing peptides in the MHC groove, triggering clonal deletion during MHC-specific thymic selection. However, FG-loop cysteines do not interfere with MHCi thymic selection as native cell surface proteins, ligands for MHCi TCR, contain disulfide-linked rather than free cysteines^6,8^. Although less dramatic than the clonal deletion of FG-loop cysteines, FG-loop usage of the hydrophobic amino acids leucine and tryptophan increased the avidity of TCR-MHC interactions, promoting clonal deletion during MHC-specific thymic selection.

While MHC-specific selection first occurs in the thymus, it is likely re-enforced and refined in the periphery. By identifying sequences common between pre-selection and mature TCR repertoires, we followed the impact of MHC-imposed constraints (i.e. restricted CDR3 length and amino acid usage) on post-selection TCRs in the periphery. Because peripheral expansion is dependent on intermittent TCR signaling stimulated by engagement of peripheral ligands, we considered increased peripheral frequency to reflect ‘peripheral fitness’ and found that the most peripherally fit TCRs displayed precisely the same optimal CDR3 length (8–13 aa) and the same restricted amino acid usage that we found during MHC-specific thymic selection. Further, the peripherally fit TCRs were often found in multiple mice, known as ‘public sequences’^10,11,29^. Thus, peripherally fit TCR are likely common sequences possessing CDR3 structural characteristics that we suggest are optimal for MHC-binding. It is worth noting that CDR3 amino acid usages not only distinguish between MHCr and MHCi repertoires, but they also reveal differences between CD4 and CD8 T cells within the MHCr repertoire (Supplementary Figure 5m). It is conceivable that other mature T cell populations, such as conventional and Foxp3+ Treg T cells, may also exhibit biased repertoire properties associated with these distinct populations.

The germline model of MHC-restriction postulates that MHC-restriction is entirely imposed by germline-encoded TCR-CDR1/2 regions that have evolved to specifically bind to MHC^9,30,31^. In this view, all TCRs generated in the pre-selection repertoire could only bind to MHC and not other ligands, a germline requirement which is directly contradicted by the current study showing that the thymus selects MHC-restricted TCRs from a broader pre-selection repertoire. Moreover, it has been previously shown that MHC-restriction was preserved despite the loss of specific CDR1/2 structures^32^. Nevertheless, there is clear evidence that TCR and MHC molecules have co-evolved, but such co-evolution is insufficient to account for MHC-restriction. For example, antibodies have longer CDR3 segments than TCRs^21,33,34^ as a result of having longer recombining gene segments than TCRs, indicating that TCRs have evolved shorter CDR3 recombining gene segments in order to accommodate MHC-binding. Importantly, the present study provides compelling evidence that such TCR and MHC co-evolution is not sufficient explanation for MHC-restriction, as MHC-restriction additionally requires thymic selection.

In conclusion, the present study documents the molecular mechanism of how MHCr TCRs are selected from a broader pre-selection repertoire in developing thymocytes. The MHC-imposed sequence and structural restrictions on TCR reflect either the structural compatibility of the receptor for MHC binding or the result of limiting autoreactive sequences. Collectively, they define the criteria necessary for mature T cells to express an MHC-restricted TCR repertoire and provide the molecular basis for positive and negative selections.

## Methods

### Reagents

Monoclonal antibodies against mouse CD4 (GK1.5 or RM4.4), CD8α (53-6.7), CD44 (IM7), CD69 (H1.2F3) were purchased from Invitrogen, CD62L (MEL-14), TCRβ (H57-957), CD45R/B220 (RA3-6B2), NK1.1 (PK136) and γδTCR (GL-3) were obtained from BD Biosciences (San Jose, CA).

### Animals

C57BL/6 (B6) mice were obtained from the Frederick National Laboratory for Cancer Research. BALB/c and B10.BR mice were obtained from The Jackson Laboratory (Bar Harbor, ME). B6.Bcl2^Tg^, B2m^−/−^H-2Ab1^−/−^ (MHCKO) mice, B2m^−/−^H-2Ab1^−/^−CD4^−/−^CD8a^−/−^ (Quad^KO^) mice, and Quad.Bcl-2^Tg^ mice were bred in our own animal facility. Animal experiments were approved by the National Cancer Institute Animal Care and Use Committee, and mice were cared for in accordance with National Institutes of Health guidelines.

### Flow cytometry and cell sorting

Different T cell populations were identified by flow cytometry using LSRII or LSRFortessa (BD Biosciences). Specifically, the double negative (DN) thymocytes were first stained with biotinylated antibodies against γδTCR, CD45R/B220, NK1.1, and TCRβ, washed with MACS buffer (PBS with 0.5% BSA, 1 mM EDTA) and then incubated with Strepatavidin Microbeads, CD4 (L3T4) Microbeads and CD8 (Ly-2) Microbeads (Miltenyi Biotec). Negatively selected thymocytes were then stained with anti-CD4, CD8, and CD69 monoclonal antibodies and cell sorted using FACSAria II (BD Biosciences). Pre-selection unsignaled DP thymocytes were sorted based on CD4^+^, CD8α^+^, and CD69^−^ gates from B6 or MHC-KO animals. For purification of naïve T cells, lymph node (LN) T cells were first depleted of MHC-II^+^ and Ig^+^ B cells by antibody-mediated magnetic beads depletion (Biomag, Qiagen) and then FACS sorted for γδTCR^−^/TCRβ^+^, and CD44^−^ /CD62L^+^ populations.

### High throughput TCR repertoire sequencing

TCRβ chains were sequenced from >30 mice of different genetic background using Illumina sequencers, performed by Adaptive Biotechnologies Corp (Seattle, WA) or iRepertoire Inc. (Huntsville, AL) (Supplementary Table 1-3). In brief, pre-selection (TCR^-^) DN thymocytes were first enriched by magnetic depletion of NK1.1^+^, B220^+^, γδTCR^+^, CD4^+^, CD8β^+^, and TCRβ^+^ populations, and then sorted as CD4^−^, CD8^−^ and CD69^−^ unsignaled DN thymocytes (Supplementary Fig. 8). Pre-selection unsignaled DP thymocytes were sorted based on CD4^+^, CD8α^+^, and CD69^−^ gates from B6 or MHC-KO animals. Naïve mature LN αβT cells were obtained by magnetic depletion of Ig^+^ B cells, then FACS sorted for γδTCR^−^/TCRβ^+^, and CD44^−^ /CD62L^+^ population (Supplementary Fig. 8). A total of 5 × 10^6^–2 × 10^7^ sorted cells were washed in PBS and lysed in Trizol. RNA was extracted using the RNEasy protocol (Qiagen) and 2 μg per sample reverse transcribed to cDNA by oligo (dT) priming with the SuperScript TM III First-Strand Synthesis System (Invitrogen). Genomic DNA was removed using DNA-free kit (Ambion). The sequencing data included CDR3 nucleotide and amino-acid sequences, raw copy number (read counts), V/J genes and gene families, adjusted copy number and frequency. Sequences containing stop codons or unresolved amino acids, as well as lacking the N-terminal cysteine residue in CDR3 were rejected before analyses. While the sequencing depths varied between samples from Adaptive Biotech and iRepertoire, the results from these separate sequencing facilities are very consistent for the same strain of mice with regard to both CDR3 length distribution and Cys usage (Supplementary Fig. 9).

TCRα sequencing were carried out by either iRepertoire, Inc. or National Institute on Aging core facility^35^. The latter used primers containing unique molecular identifiers (UMI). In brief, total cellular RNA was extracted from 10^6^ sorted T cells using RNAeasy micro kit QIAcube (Qiagen). Libraries of TCRα and TCRβ were constructed from the isolated RNA (~250 ng) by RT-PCR using SMARTScribe reverse transcriptase (Clonetech) with primers specific to the constant region of mouse TCRα (Supplementary Table 4). The RT-PCR primers contain a unique molecular identifier (UMI), SmartNNNNa oligo of 12 random ‘N’ nucleotides and four dU nucleotides^36^. The first round PCR reaction was carried out using High Fidelity Platinum Taq DNA polymerase (Life Technologies) with a universal 5′-primer MISS and 3′-primers of mTRAC1. Primers for the second PCR reactions, P7MIS-nSB, contain a partial P7-adapter sequence, an 8-nucleotide sample barcode sequence, and mTRAC3. After addition of the barcodes, samples were amplified using P7-adpater primer set, purified by gel electrophoresis, and subjected to Illumina HiSeq 2500 sequencer according to manufacturer’s instruction. DNA sequences were processed to identify the UMI and sample barcode using a custom python script, mapped and assembled to TCRα gene and CDR3 using MIGEC^36^. Despite of using different sequencing technologies and platforms, the results are consistent for the same strain of mice regarding both CDR3 length distribution and Cys usage (Supplementary Fig. 9).

### Analyses of TCR sequences

Sequences corresponding to the FG-loop region of CDR3 were identified through their alignment on the tertiary structures of TCR. Majority of repertoire analyses were performed using in-house TCR repertoire analysis software while some were performed using ImmunoSEQ software from Adaptive Biotechnologies (http://www.adaptivebiotech.com/immunoseq). Amino acid compositions were calculated using either ProtParam of ExPASy (web.expasy.org/protparam) or in house software. The amino acids and sequence length comparison figures and their statistics were generated using GraphPad Prism 6 (www.Graphpad.com, La Jolla, California, USA). TCRβ CDR3 region is defined as residues between Cys 104 and Phe 118, and are numbered from 105 to 117, with corresponding FG-loop between 108 and 114. For CDR3 shorter or longer than 13 residues deletions and insertions were introduced according to the International ImMunoGeneTics information system (IMGT) convention^14^. Sequences corresponding to mouse antibody heavy chain CDR3H were compiled through sequence search of non-redundant protein sequence databases using BLAST (Basic Local Alignment search tool, blast.ncbi.nlm.nih.gov/Blast.cgi). The clonal diversity of each sequence repertoires were estimated using an Inverse Simpson Index 1/Σ (Nu/Nt)^2^ where Nt and Nu are total number of reads and unique number of reads associated with an individual unique sequence^17^. Hence, Nu/Nt represents the frequency of individual unique sequences. Pearson correlation coefficient (*r*) between *n* sets of covariants *x*, *y* is calculated with an equation:

$$r = \left[ {{n}\left( {\Sigma {xy}} \right)-\left( {\Sigma {x}} \right)\left( {\Sigma {y}} \right)} \right]/\mathrm{sqrt}\left\{\left[ {{n}\Sigma {x}^2-\left( {\Sigma {x}} \right)^2} \right]\left[ { {n}\Sigma {y}^2-\left( {\Sigma {y}} \right)^2} \right]\right\}$$

### Reporting summary

Further information on experimental design is available in the Nature Research Reporting Summary linked to this article.

## Supplementary information

Supplementary Information

Peer Review File

Reporting Summary

## Data Availability

The sequence data described in the manuscript are freely accessible through ImmuneAccess [https://clients.adaptivebiotech.com/pub/lu-2019-natcomms] with doi [10.21417/JL2019] and are available upon request.
