# Supplementary material for: Molecular constraints on CDR3 for thymic selection of MHC-restricted TCRs from a random pre-selection repertoire
Source: Nat Commun. 2019 Mar 4;10:1019. doi: 10.1038/s41467-019-08906-7 (PMC6399321; doi:10.1038/s41467-019-08906-7)
Supplement: Supplementary file 1 — Supplementary Information [file 41467_2019_8906_MOESM1_ESM.pdf]

Supplementary Information

Molecular constraints on CDR3 for thymic selection of MHC-restricted TCRs from a random pre-selection repertoire

Supplementary Figure 1

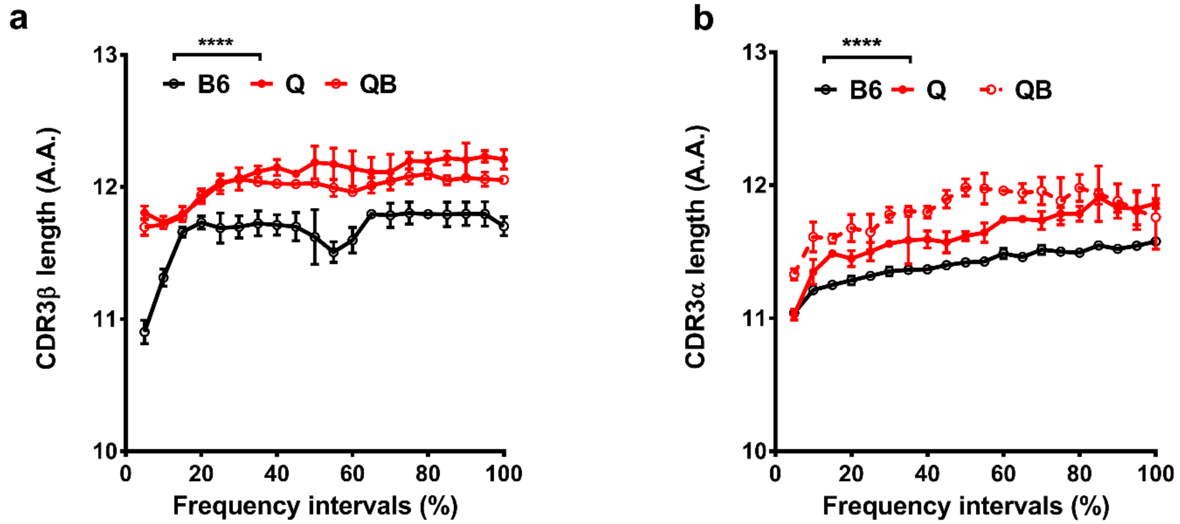

Supplementary Figure 1. Frequency-dependent CDR3 length distributions. The distribution of average length of CDR3β (a) and CDR3α (b) in B6, Q and QB repertoires with respect to their frequency intervals. Sequences from three mice of each strain were grouped by 5% intervals from the top 5% frequent sequences until the accumulation of all sequences (100%).

Supplementary Figure 2

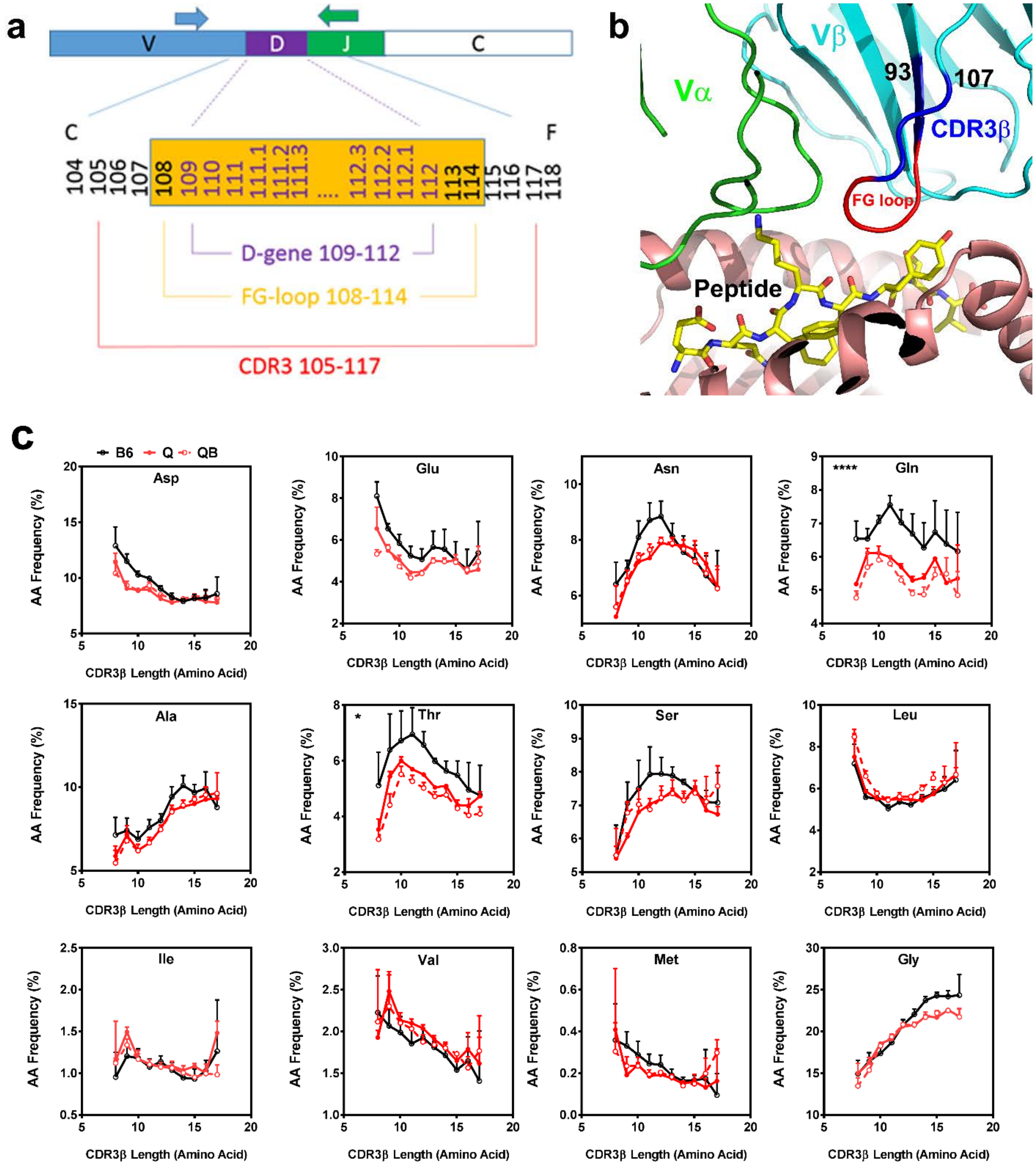

Supplementary Figure 2. TCR-CDR3 amino acid compositions in B6, Q and QB repertoires. (a) Schematic diagram showing the region of CDR3 amino acids in D gene and FG-loop. (b) the structure of CDR3β FG loop in a representative TCR-pMHC complex (PDB ID: 1OGA). **(c)** Frequencies of individual amino acids in CDR3β from representative B6, Q and QB repertoires with respect to their CDR3 length.

Supplementary Figure 2

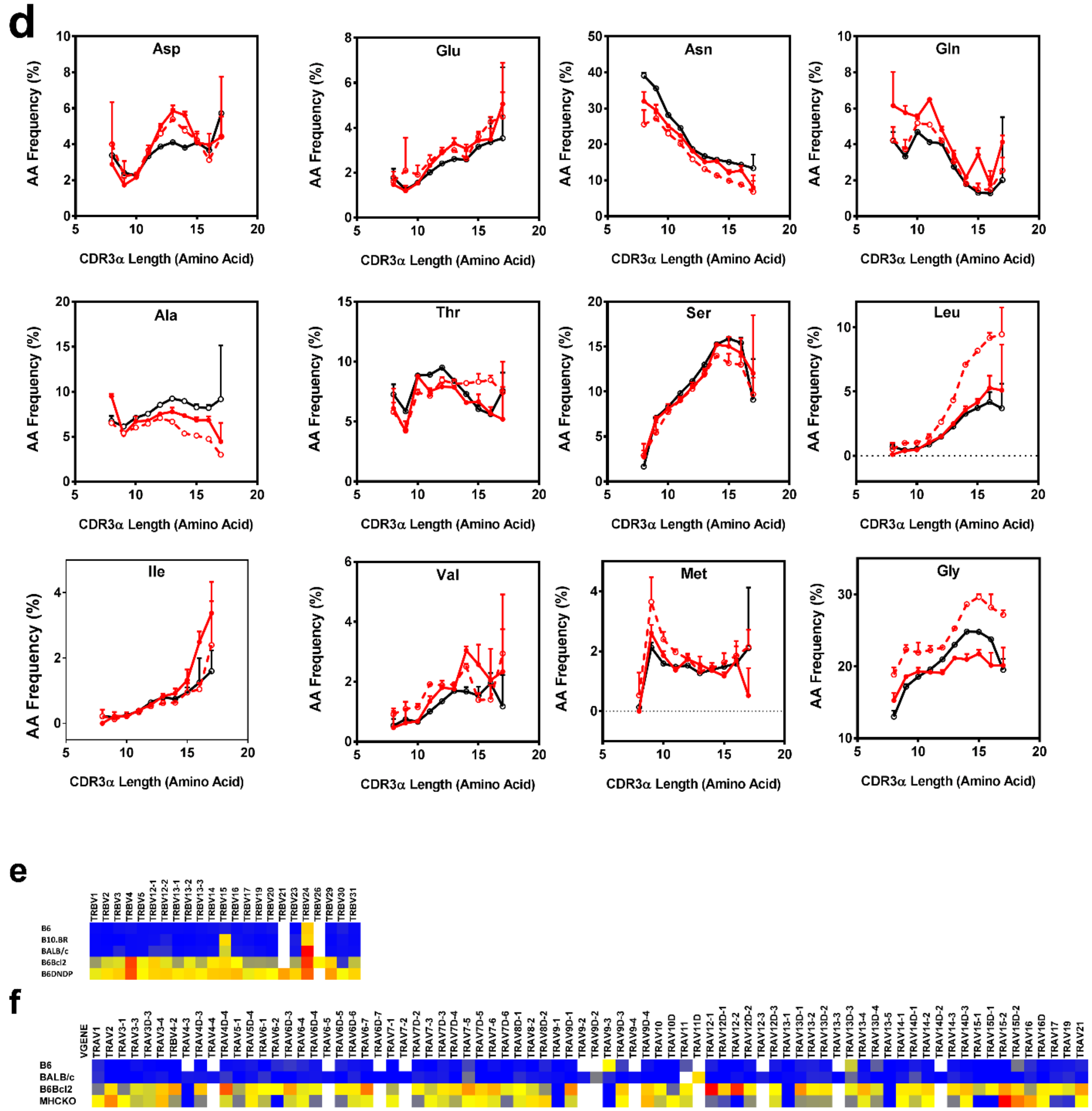

Supplementary Figure 2. (d) Frequencies of individual amino acids in CDR3α from representative B6, Q and QB repertoires with respect to their CDR3 length. (e-f). Frequencies of Cys residue in CDR3β (e) and CDR3α (f) FG-loops displayed as a heat map for various repertoires with respect to each germline V-gene.

Supplementary Figure 3

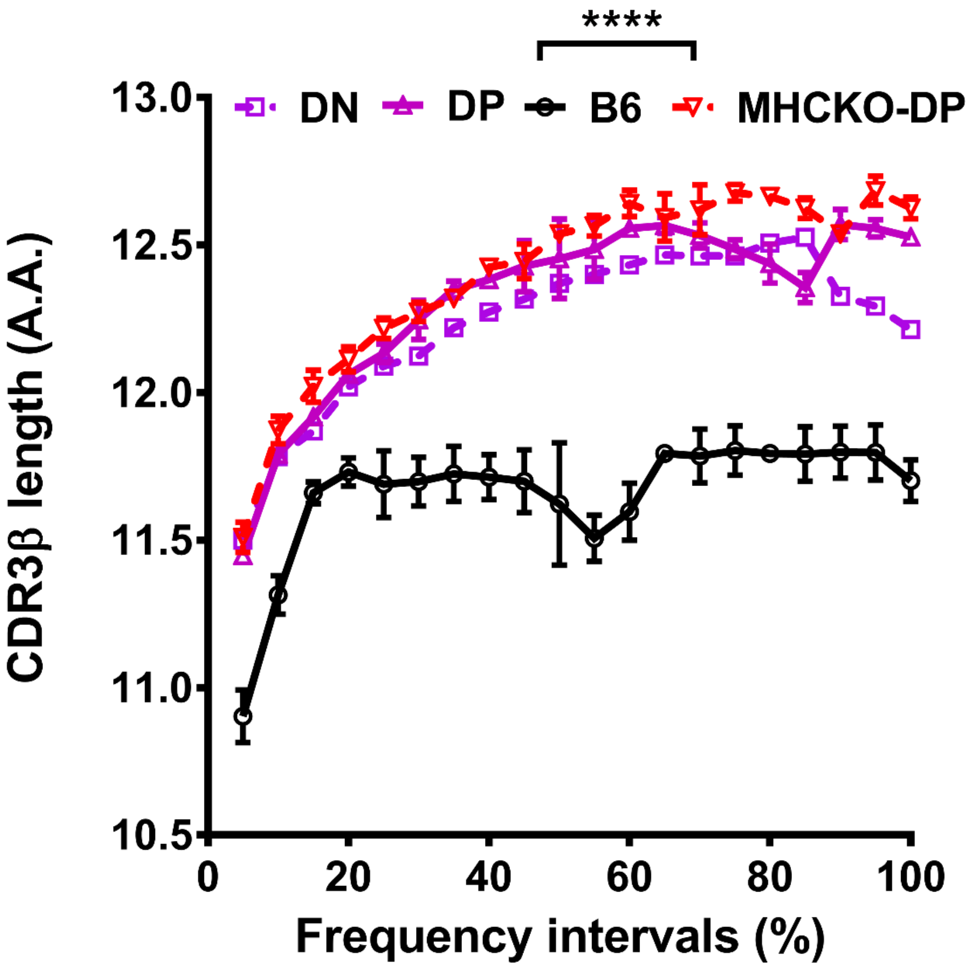

Supplementary Figure 3. CDR3β length distributions in pre-selection repertoires. The distribution of average length of CDR3β in various pre-selection repertoires compared to B6 with respect to their frequency intervals. Sequences from three mice of each strain were grouped by 5% intervals from the top 5% frequent sequences until the accumulation of all sequences (100%).

Supplementary Figure 4

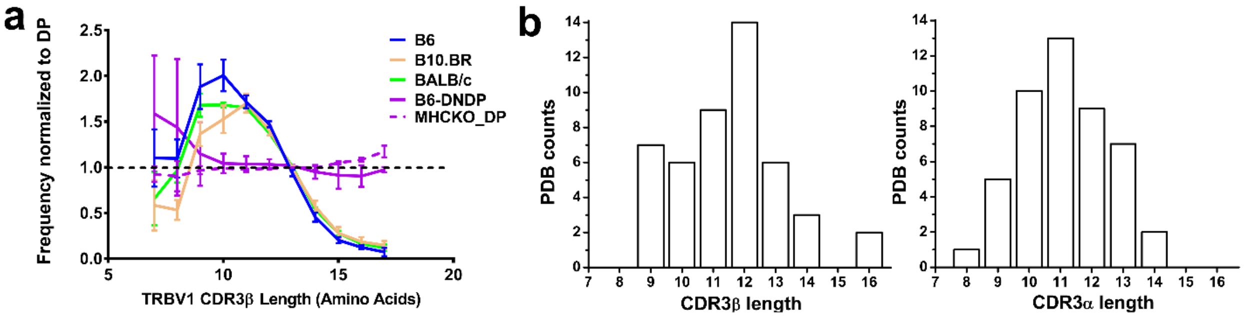

Supplementary Figure 4. CDR3 length preference. (a). Comparisons of normalized CDR3β length distributions of TRBV1 gene-containing sequences between MHCr and pre-selection repertoires. The preference of CDR3β length distributions is indicated by respective fold changes to B6 DP_1. (b). CDR3β and CDR3α length distributions of 55 MHC-restricted αβ TCRs structures from Protein Data Bank (PDB).

Supplementary Figure 5

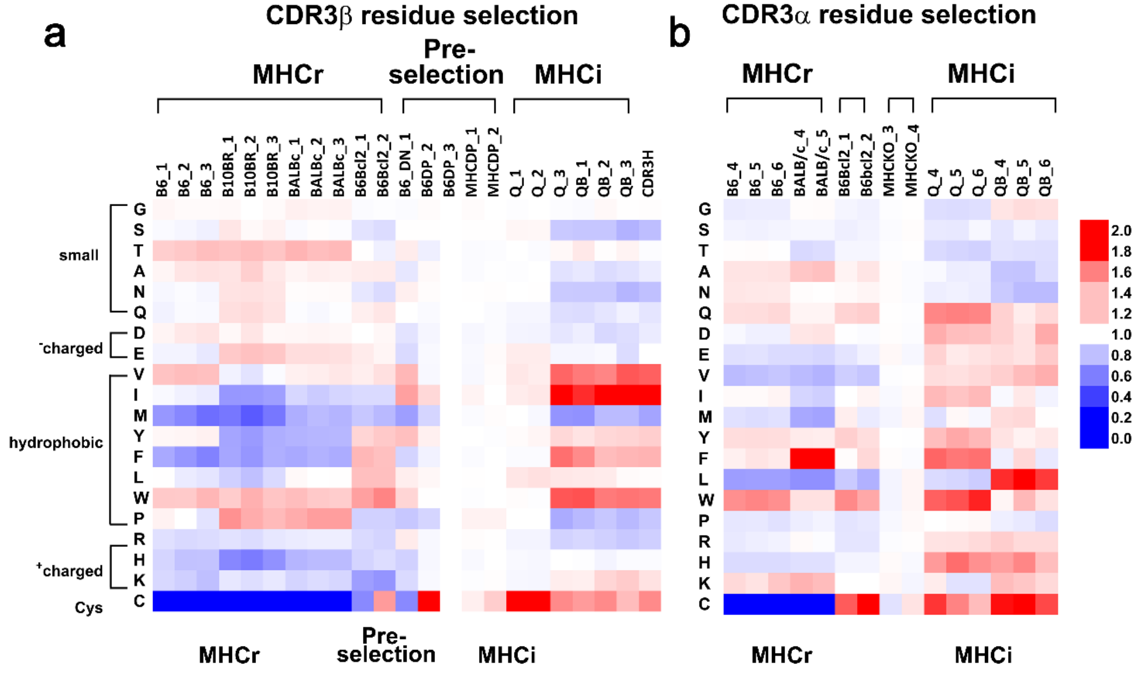

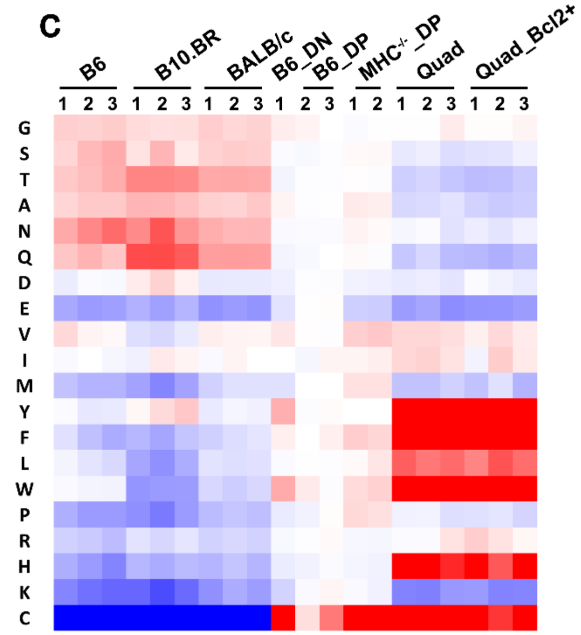

d

		Forward 5'-3' coding	Complementary 3'-5' coding
TRBD1*01		GGGACAGGGGGC	GCCCCCTGTCCC
Amino acids	Frame 1:	G T G G	A P C P
	Frame 2:	G Q G	P P V
	Frame 3:	D R G	P L S
TRBD2*01		GGGACTGGGGGGGC	GCCCCCCCAGTCCC
Amino acids	Frame 1:	G T G G	A P P V
	Frame 2:	G L G G	P P Q S
	Frame 3:	D W G G	G G S G

e

Amino Acids	Number of D-gene Codons	Coding Frequency (%)
G	17	38.6
S	3	6.8
T	2	4.5
A	2	4.5
N	0	0
Q	2	4.5
D	2	4.5
E	0	0
V	2	4.5
I	0	0
M	0	0
Y	0	0
F	0	0
L	2	4.5
W	1	2.3
P	9	20.5
R	1	2.3
H	0	0
K	0	0
C	1	2.3

Supplementary Figure 5. Amino acid usage in TCR CDR3 FG-loop from MHCr, MHCi and pre-selection repertoires. (**a,b**) Individual amino acid usage of CDR3β FG-loop (**a**) and CDR3α FG-loop (**b**) displayed as fold changes with respect to those in B6 DP1 repertoire in heat maps. (**c**) Amino acid frequencies in D-gene region of CDR3β FG-loop displayed as a heatmap with fold changes compared to those in B6 DP1 repertoire. (**d,e**)Coding sequences of Dβ genes and their resulted frequencies of each amino acid.

Supplementary Figure 5

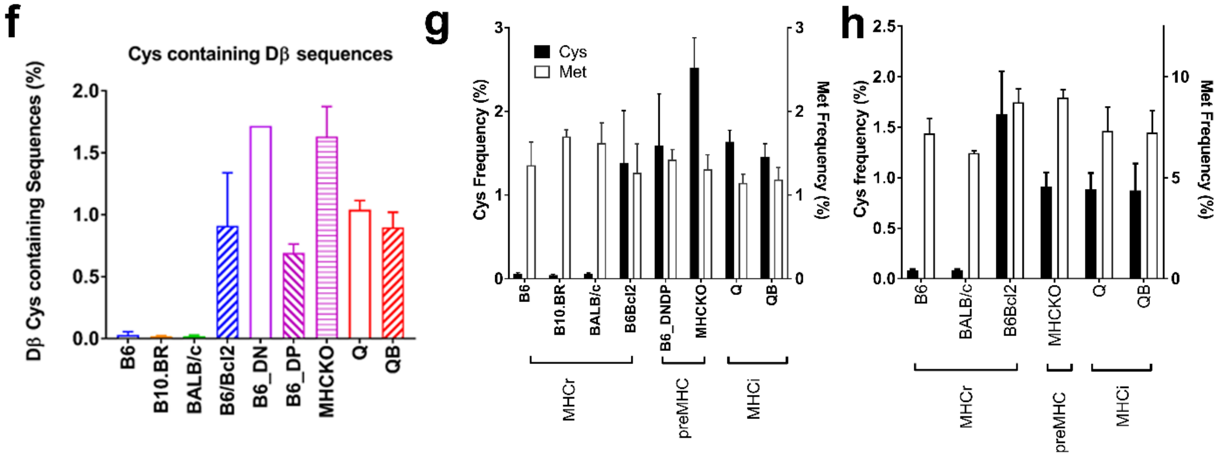

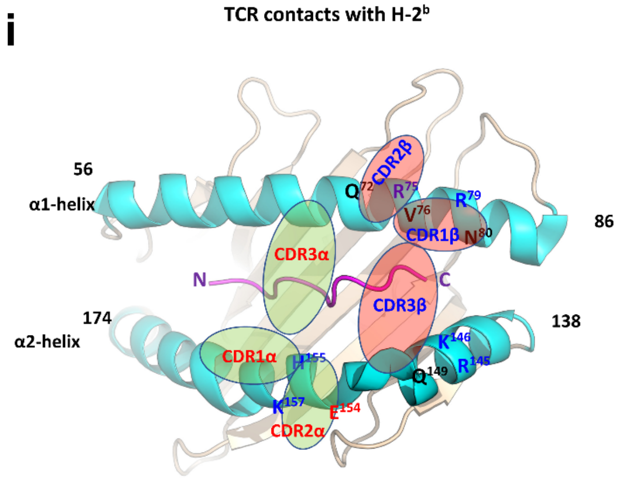

TCRβ contacting MHC-I residues			TCRα contacting MHC-I residues		
CDR1β	CDR2β	CDR3β	CDR1α	CDR2α	CDR3α
V76	Q72	R145/H	H155/R	E154	P4-P6
N80/T	R75	K146		K157	
	R79	Q149			
		P6-P8			

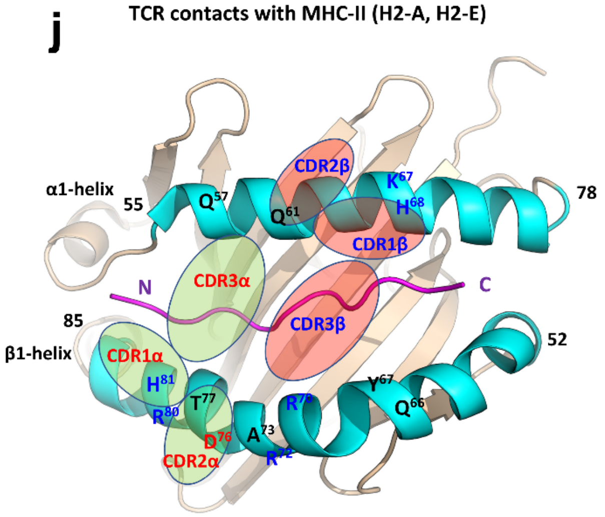

TCRβ contacting MHC-II residues			TCRα contacting MHC-IIa residues		
CDR1β	CDR2β	CDR3β	CDR1α	CDR2α	CDR3α
Q61/A	Q57	Q66/E	T77	R72	Q57
H68/A	K67	Y67	H81	A73	Q61/A
		R70/K		D76	P3-P4
		P4-P6		R80	

Supplementary Figure 5. (**f**) Percentage of sequences containing Cys in CDR3β D regions observed in various repertoires. (**g,h**) Amino acid usage comparison of sulfur-containing residue Cys and Met in CDR3β FG loop (**g**) and CDR3α FG loop (**h**) among various repertoires. (**i,j**) Putative TCR interaction footprint on H-2D^b and K^b (MHC-I), as well as on H2-A and H2-E (MHC-II) molecules based on published structures of TCR-MHC complexes in canonical docking orientation. The approximate positions of six CDR loops are indicated with oval shapes and their contacting MHC residues are listed.

Supplementary Figure 5

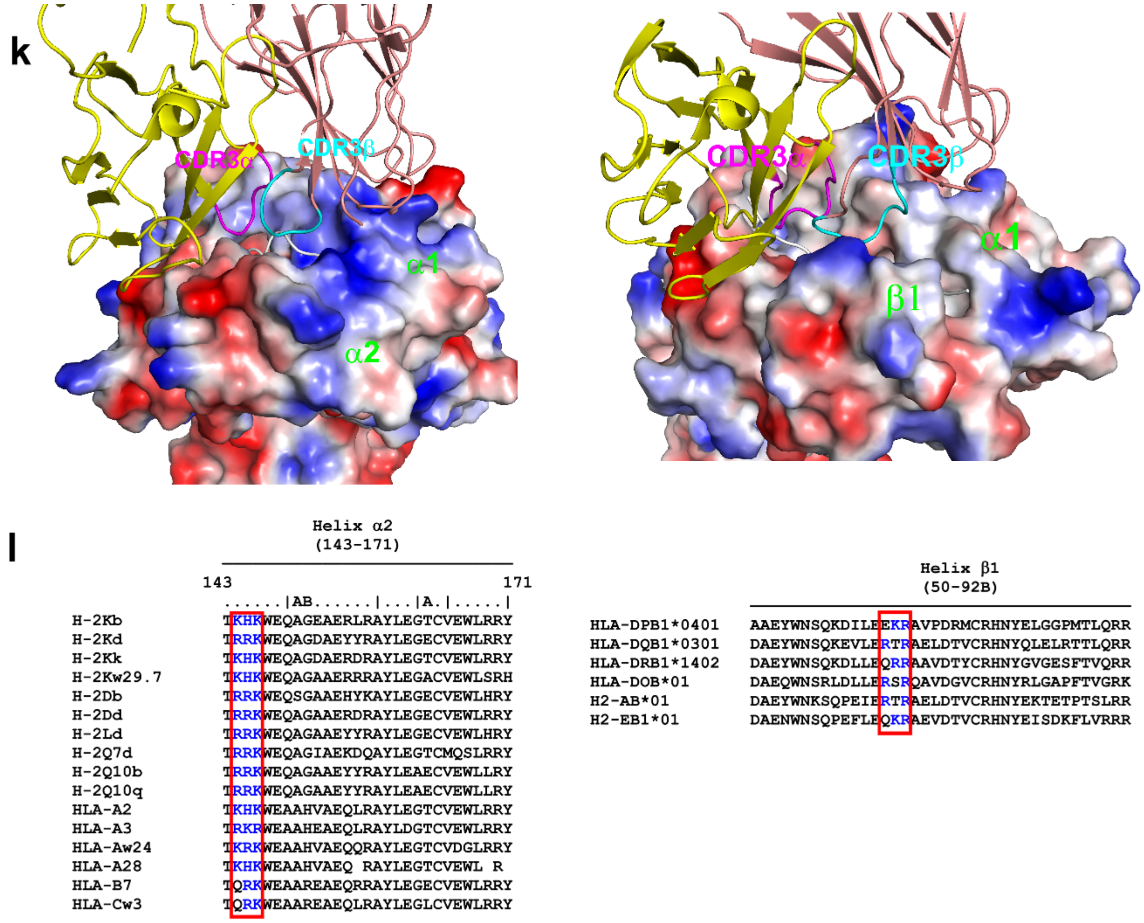

Supplementary Figure 5. (**k**) Crystal structure of one representative TCR/MHC-I (PDB ID: 1OGA) and TCR/MHC-II complex (PDB ID: 2PXY). The TCR beta and alpha chain were shown in cartoons colored in pink and yellow, respectively. The MHC molecules were represented by electrostatic surfaces with negatively charges colored in blue and positively charged residues colored in red, respectively. (**l**) Sequence alignment of helix α2 of MHC-1 alleles and helix β1 of MHC-II alleles. The negatively charged motifs on helix α2 and β1 were highlighted in red box.

Supplementary Figure 5

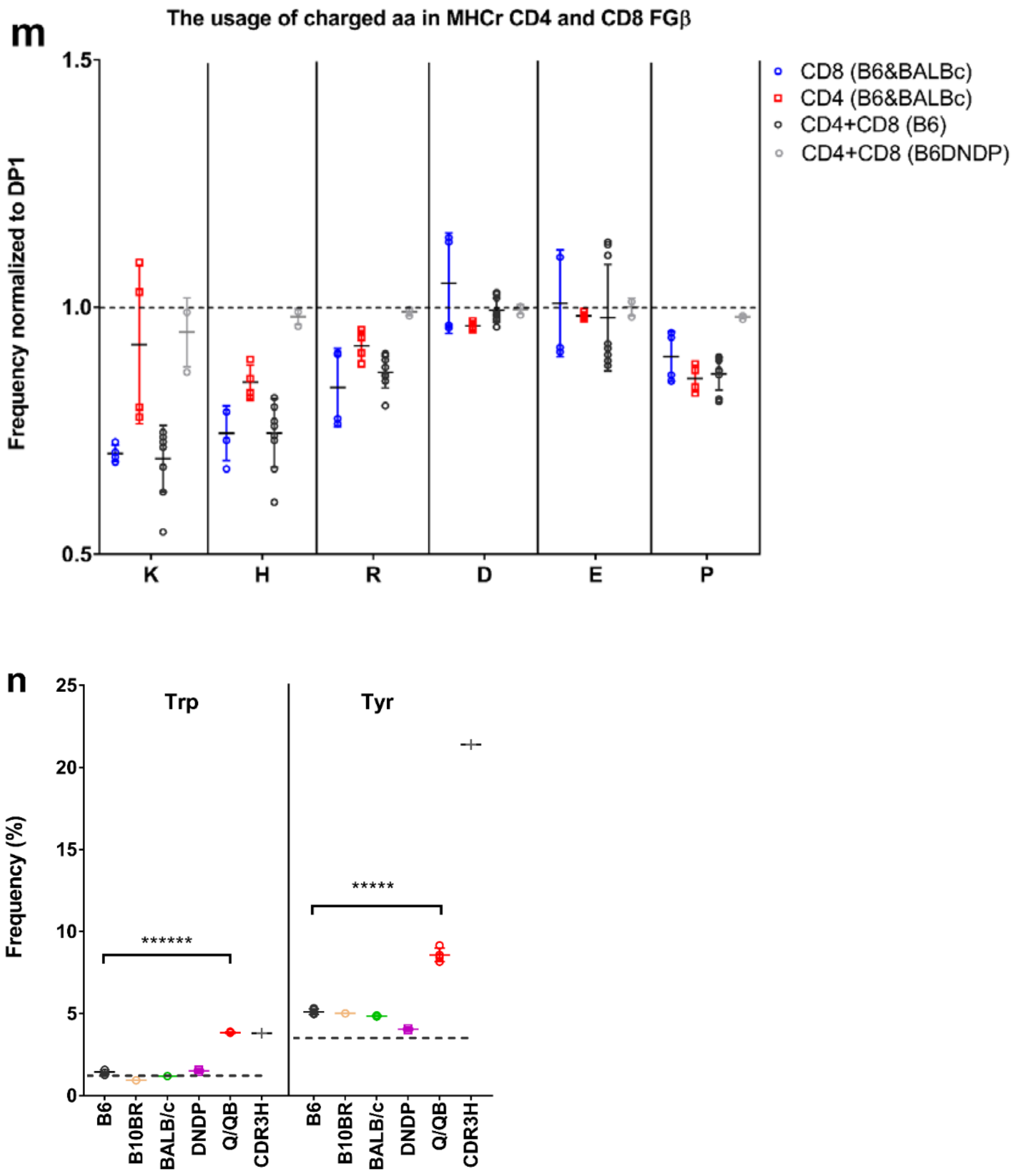

Supplementary Figure 5. (**m**) The comparison of amino acid usage of residues K, H, R, D, E, P in CDR3β FG loop between MHC I restricted CD8 T cells, MHC II restricted CD4 T cells, mixed TCRs (CD8 and CD4 T cells) and pre-selection TCRs. (**n**) Observed frequencies of Trp and Tyr in the public CDR3β FG-loop of MHCr, MHCi as well as pre-selection repertoires. The TCRβ sequences are public sequences of each strain, namely common sequences observed in at least three animals of their individual strains. As a comparison, their respected frequencies in mammalian proteins are indicated by dashed lines and their frequency in antibody CDR3H are indicated by (+).

Supplementary Figure 6

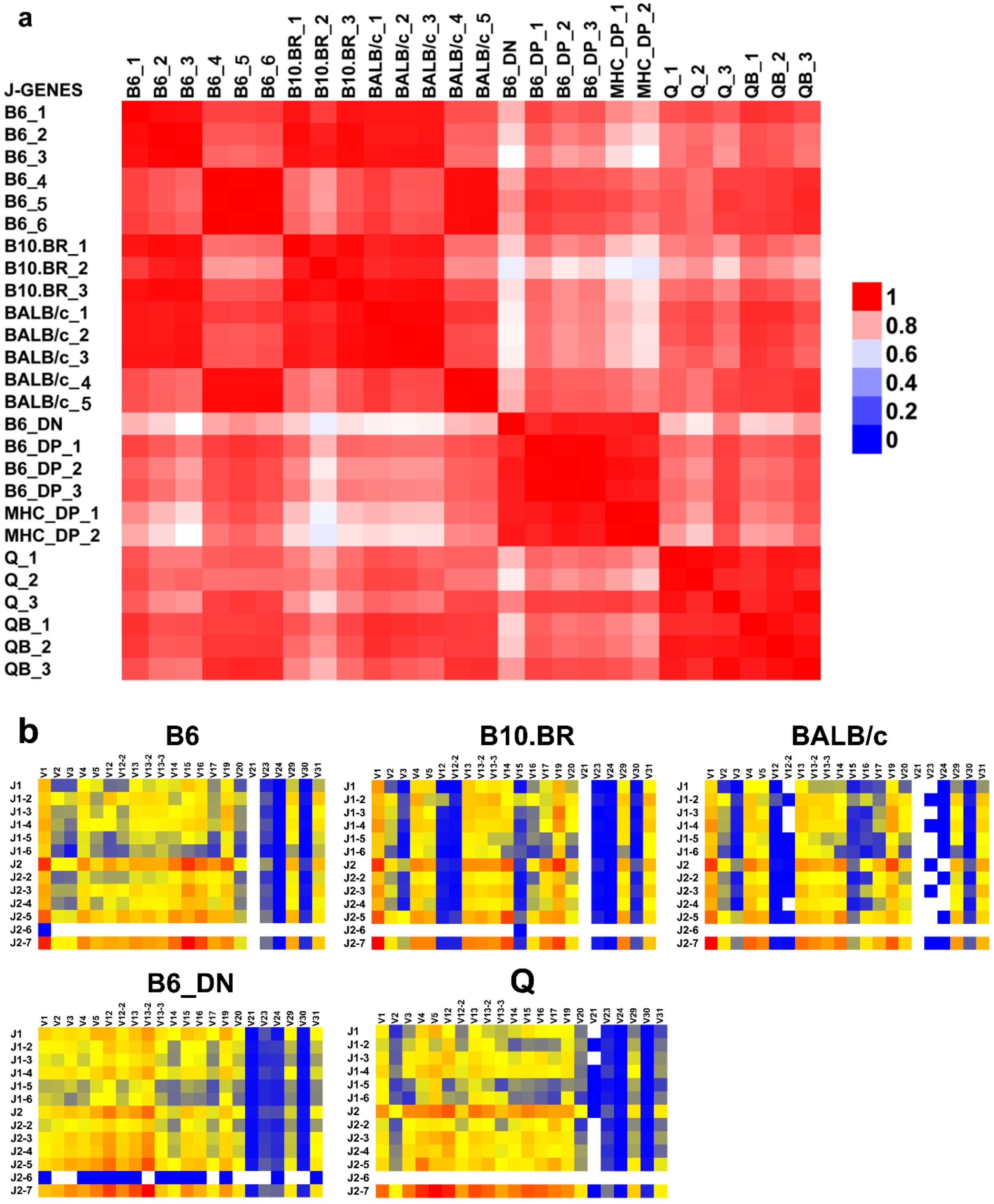

Supplementary Figure 6. Analysis of V- and J- gene pairing. (**a**) Heatmap display of Pearson correlation coefficients calculated using Jβgene usage among pairwise repertoire sequences for TCRβ. (**b**)Representative Vβ and Jβ paring in various repertoires.

Supplementary Figure 6

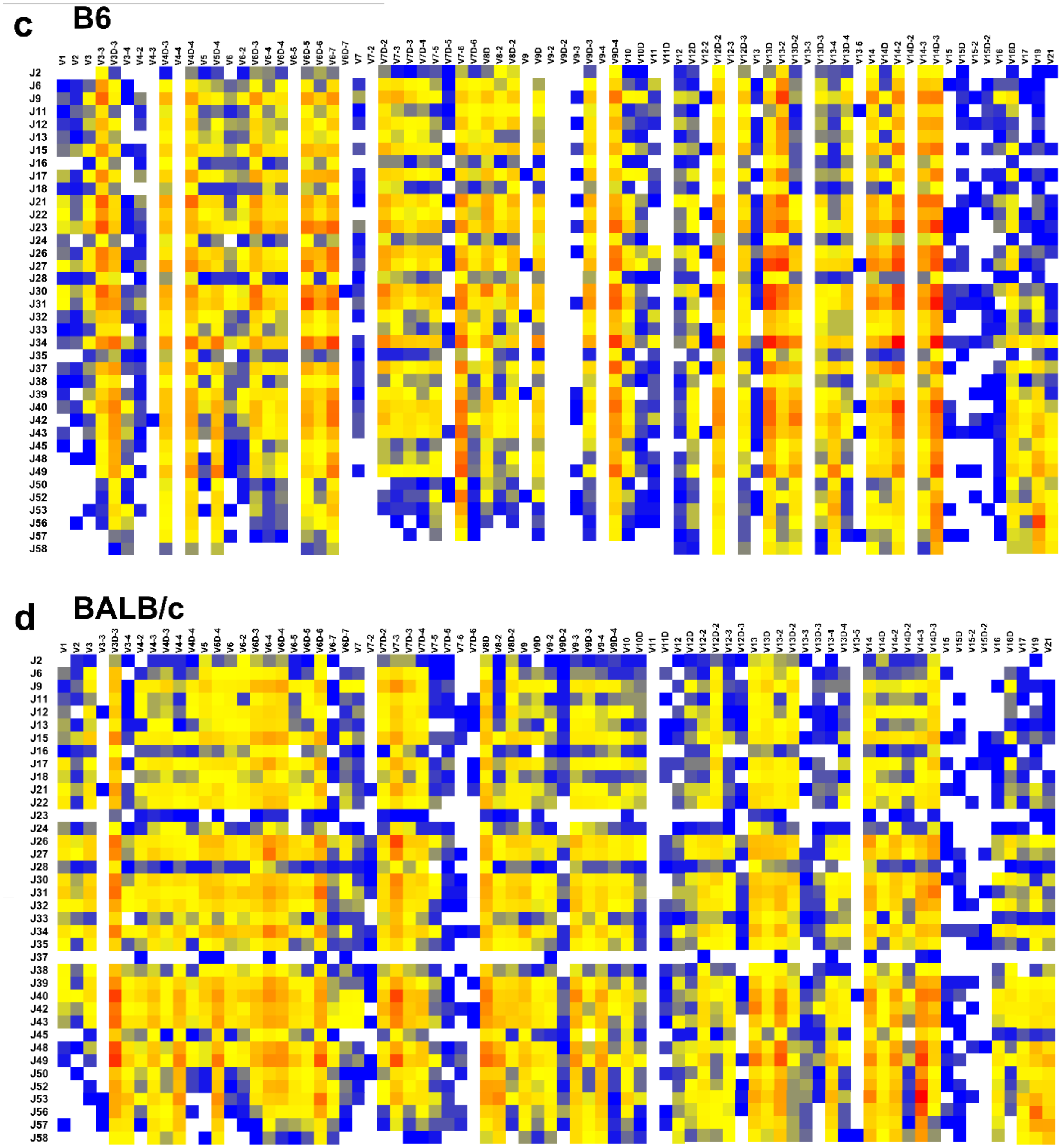

Supplementary Figure 6. (**c-d**) Representative Vα and Jα paring in various repertoires.

Supplementary Figure 6

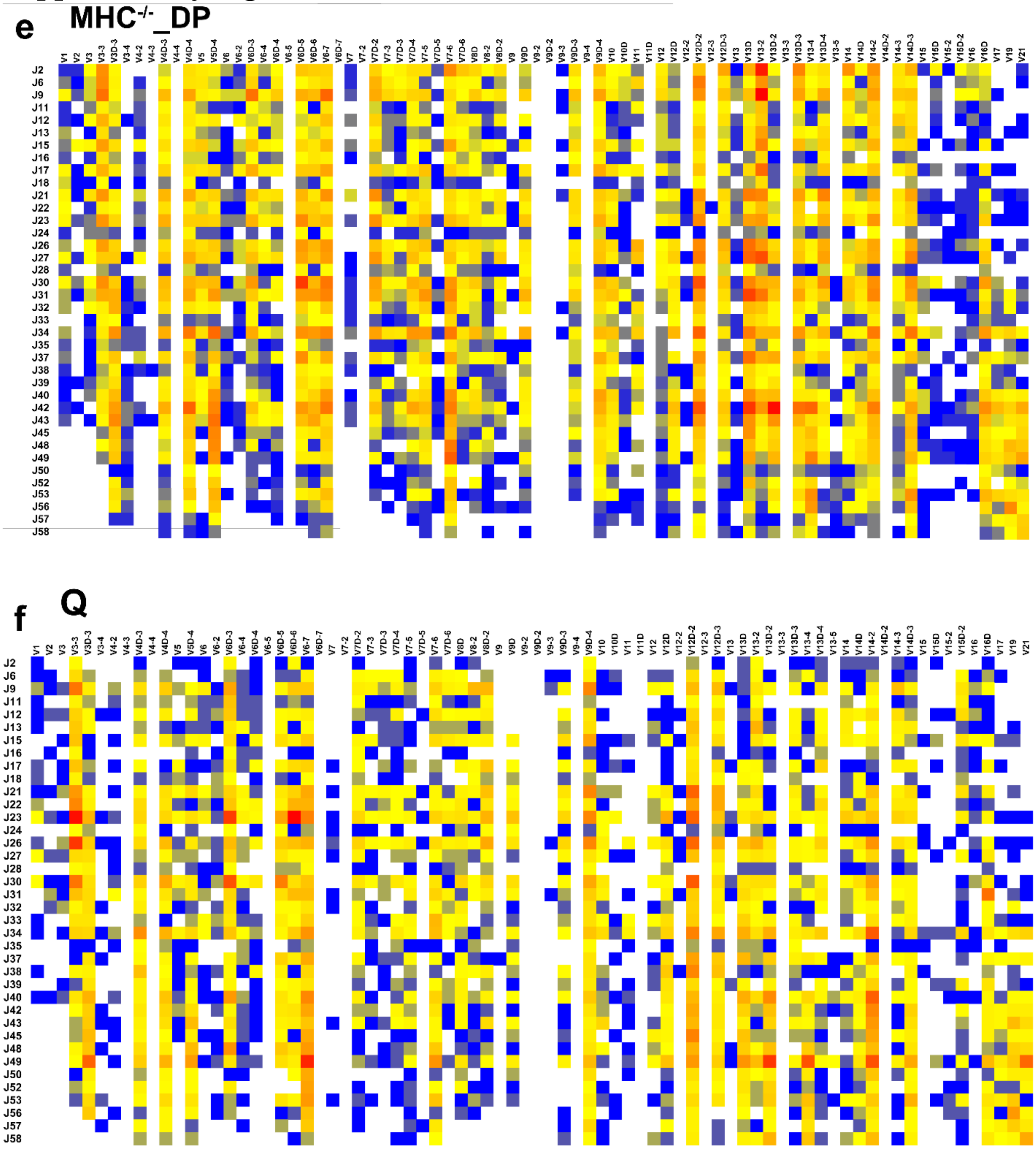

Supplementary Figure 6. (**e-f**) Representative Vα and Jα paring in various repertoires

Supplementary Figure 6

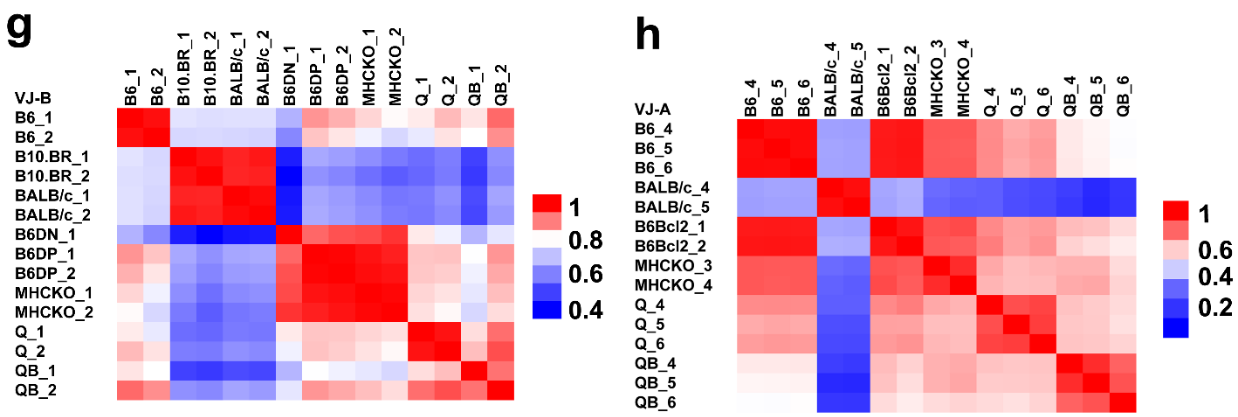

Supplementary Figure 6. (**g-h**) Heatmap display of Pearson correlation coefficients calculated using VJ pairing frequencies among pairwise repertoire sequences for TCRβ (**g**) and TCRα (**h**). The sequences of Vβ and Jβ were from Adaptive Biotechnology and those of Vα and Jα were from Irepertoire.

Supplementary Figure 7

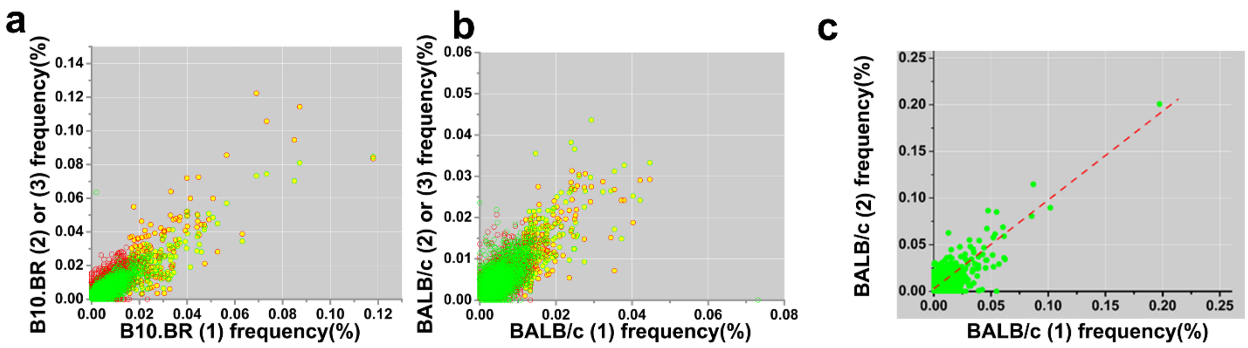

Supplementary Figure 7. Frequency scatter plot and cysteine usage. Frequency scatter plots between individual (**a**) B10.BR and (**b**) BALB/c TCRβ sequences, as well as between individual (**c**) BALB/c TCRα sequences.

Supplementary Figure 8

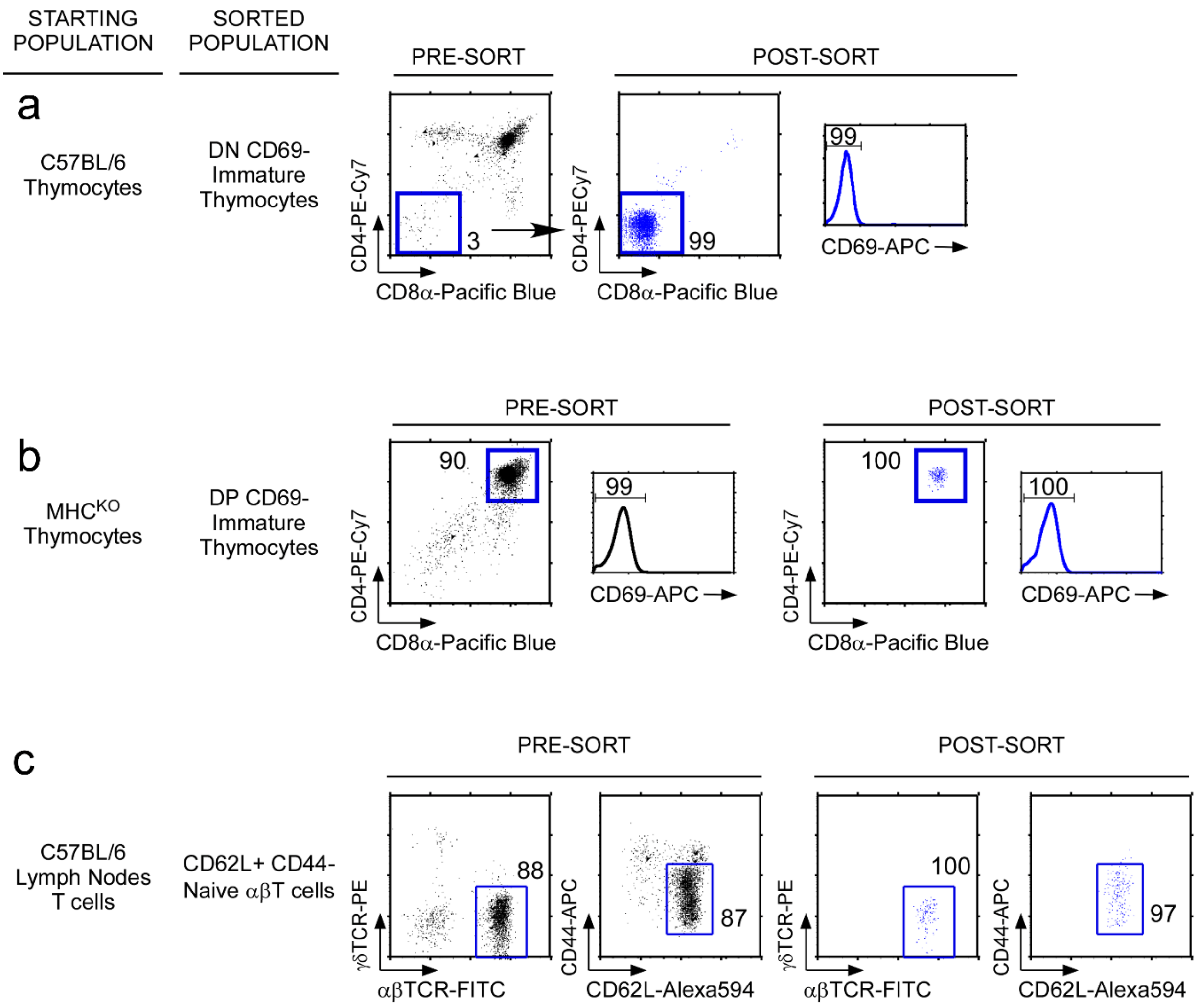

Supplementary Figure 8. Isolation procedures and FACS gating strategies for various T cell populations. (**a**) DN thymocytes were first enriched through magnetic depletion of NK1.1$^+$,B220$^+$, γδTCR$^+$, CD4$^+$, CD8β$^+$ and TCRβ$^+$ populations, and then FACS sorted as CD4$^-$,CD8$^-$ and CD69$^-$ unsignaled DN thymocytes. (**b**) The pre-selection unsignaled DP thymocytes were sorted as CD4$^+$, CD8α$^+$, and CD69$^-$ thymocytes from B6 or MHC-KO animals. (**c**) Naïve mature LN αβT cells were obtained by first magnetic depleting Ig$^+$ B cells, then FACS sorting based on γδTCR$^-$/TCRβ$^+$, and CD44$^-$ /CD62L$^+$ gates.

Supplementary Figure 9

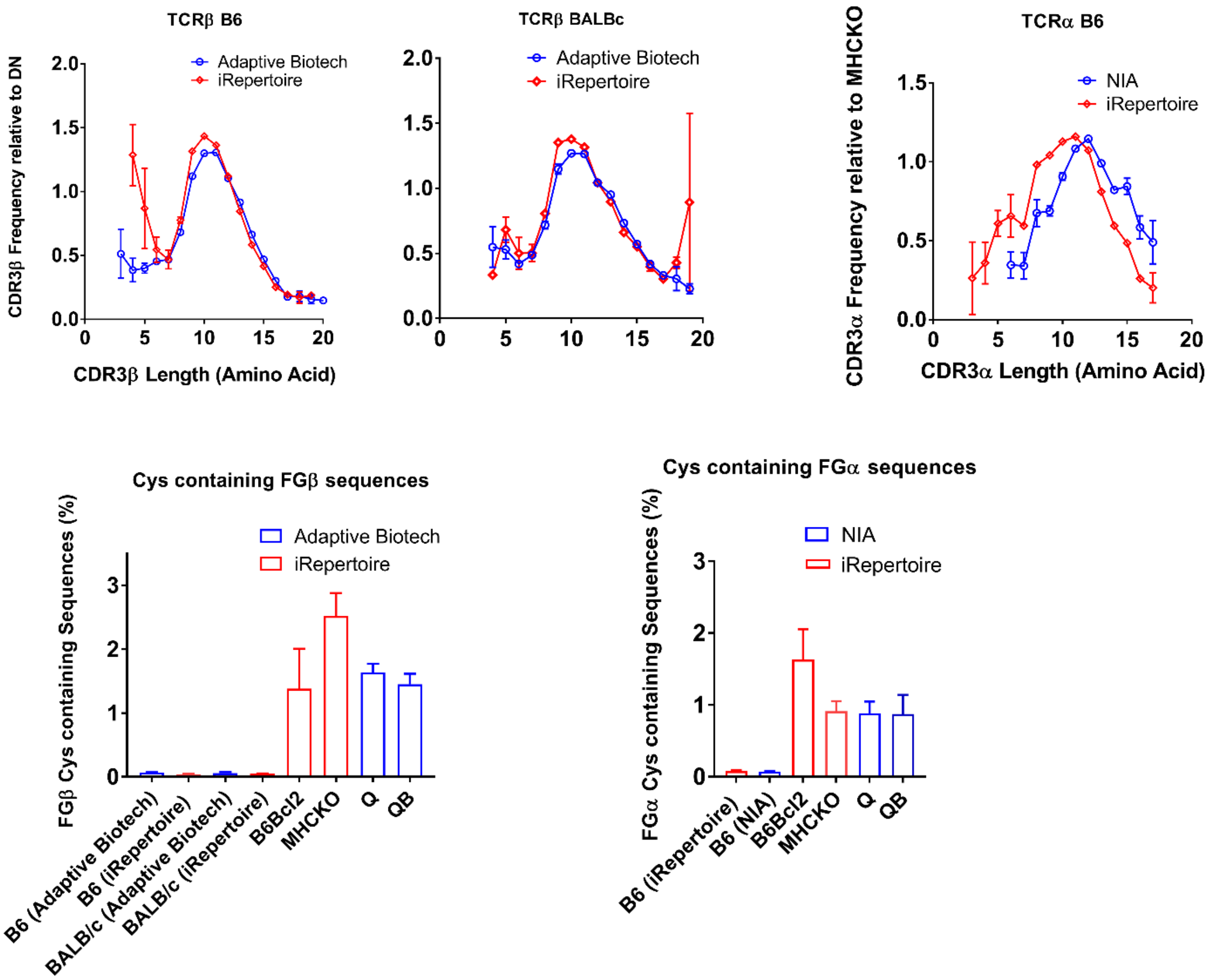

Supplementary Figure 9. Sequencing consistency among different facilities. Sequencing data from different facilities were analyzed for CDR3 length distributions and Cysteine frequency.

Supplementary Table 1. Statistics of TCRβ RNA-seq Deep Sequencing.

Strains	Sample ID	Total Sequence	Productive Total	Productive Unique	Data Set Name
**B6**	**1**	767420	721518	196260	B6_1_May2015
	**2**	295714	281123	80249	B6_2_May2015
	**3**	150775	143030	42919	B6_3_May2015
	**4**	2526522	2348572	506744	B6_09132016_1
	**5**	2545728	2361384	512742	B6_09132016_2
	**6**	2153443	2009768	457733	B6_09132016_3
	**4[1,2]**	867737	831401	64446	B6_09132016_1B
	**5[1,2]**	897749	863290	59008	B6_09132016_2B
	**6[1,2]**	893406	863758	66979	B6_09132016_3B
**B10.BR**	**1**	578131	524473	133416	B10-BR_1_May2015
	**2**	198631	182303	50338	B10-BR_2_May2015
	**3**	743225	681319	171824	B10-BR_3_May2015
**BALB/c**	**1**	429684	423303	123315	BALB-c_1_May2015
	**2**	574962	561061	157835	BALB-c_2_May2015
	**3**	483834	474277	137844	BALB-c_3_May2015
	**4**	2311699	2246098	492161	Balbc_09132016_1
	**5**	2043853	2000648	448996	Balbc_09132016_2
	**4[1,2]**	920217	888903	53811	Balbc_09132016_1B
	**5[1,2]**	910056	879428	60242	Balbc_09132016_2B
**B6Bcl-2^{tg}**	**1[2]**	811748	773940	65791	B6Bcl2_11212016_1B
	**2[2]**	800070	760839	62852	B6Bcl2_11212016_2B
**B6_DN**	**1**	12356746	11048743	276883	B6_DN_Jan2016
**B6_DP**	**1**	6873852	6145550	891818	B6_DP1_Jan2016
	**2**	8502435	7583755	885404	B6_DP2_Jan2016
	**3**	8577715	7599052	993698	B6_DP3_Jan2016
**MHC$^{-/-}$_DP**	**1**	9216625	8265749	771383	MHCKO_DP1_Jan2016
	**2**	10663932	9405971	645150	MHCKO_DP2_Jan2016
	**3[2]**	773890	735346	51206	MHCKO_11212016_1B
	**4[2]**	710989	675083	64745	MHCKO_11212016_2B
**Quad^{KO}**	**1**	946156	855585	86573	Quad_1_May2015
	**2**	541729	504949	68995	Quad_2_May2015
	**3**	982737	899700	84129	Quad_3_May2015
**Quad^{ko} Bcl-2^{tg}**	**1**	416744	387358	75584	Quad_Bcl2+_1_May2015
	**2**	471849	450045	80004	Quad_Bcl2+_2_May2015
	**3**	560416	529155	65441	Quad_Bcl2+_3_May2015

Note: [1] Samples from same animal ID are sequenced by Adaptive Biotechnology and Irepertoire.
[2] Sequences are from Irepertoire.

Supplementary Table 2. Statistics of TCRα RNA-seq Deep Sequencing.

Strains	Sample ID	Total Sequence	Productive Total	Productive Unique	Data Set Name
**B6**	**4**	811024	70864	71340	B6_09132016_1A
	**5**	820900	728665	66772	B6_09132016_2A
	**6**	816859	724718	72538	B6_09132016_3A
**B6Bcl-2^{tg}**	**1**	708082	621726	64740	B6Bcl2_11212016_1A
	**2**	739459	650274	67491	B6Bcl2_11212016_2A
**BALB/c**	**4**	826173	742551	64732	Balbc_09132016_1A
	**5**	830121	751695	66797	Balbc_09132016_1A
**MHC$^{-/-}$_DP**	**3**	667576	420127	21986	MHCKO_11212016_1A
	**4**	555507	347495	21961	MHCKO_11212016_2A
**Quad^{ko}**	**4**	1572510	1341778	11475	Q_03192018_1A
	**5**	1112323	957769	8174	Q_03192018_2A
	**6**	1583127	1348255	11546	Q_03192018_3A
**Quad^{ko}Bcl-2^{tg}**	**4**	2097719	1809892	15544	QB_03192018_1A
	**5**	5393615	4577467	39335	QB_03192018_2A
	**6**	1319935	1143107	9948	QB_03192018_3A

Note: All TCRα sequences are from Irepertoire.

Supplementary Table 3. Mice used in TCR repertoire sequencing.

Mice Strain	Number of mice	Mature Samples		Pre-selection Samples	
		TCRβ	TCRα	TCRβ	TCRα
B6	13	9	6	4	
B10.BR	3	3			
BALB/c	5	7	2		
B6Bcl-2^{tg}	2	2	2		
MHC$^{-/-}$	4			4	2
Quad^{ko}	6	3	3		
Quad^{ko}Bcl-2^{tg}	5	3	3		

Supplementary Table 4. Primers used for mouse αβTCR library and sequencing

Step	Name	5' / 3'	Sequence
**cDNA synthesis**	mTRAC1	3'	GGCGTTGGTCTCTTTGAAG
	mTRBC1	3'	CACTTGTCCTCCTCTGAAAG
	SmartN oligo	5'	AAGCAGUGGTAUCAACGCAGAGUNNNNUNNNNUNNNNUCTTrGrGrGrGp
**PCR**			
1st round	M1SS	5'	AAGCAGTGGTATCAACGCA
	mTRAC2	3'	CGGCACATTGATTTGGGAG
	mTRBC2	3'	TGTGGACCTCCTTGCCATTC
2nd round	P7M1S-n	5'	CGTGTGCTCTTCCGATC$(N)_{1-2}$(8 bp barcode)CAGTGGTATCAACGCAGAG
	mTRAC3	3'	AGGTTCTGGGTTCTGGATG
	mTRBC3	3'	GGTGGAGTCACATTTCTCAG
3rd round	P7	5'	CAAGCAGAAGACGGCATACGAGATGTGACTGGAGTTCAGACGTGTG CTCTTCCGATC
	mP5TA	3'	AATGATACGGCGACCACCGATCGTCGAGGTTCTGGGTTCTGGATG
	mP5TB	3'	AATGATACGGCGACCACCGATCGTCGGGTGGAGTCACATTTCTC
**Sequencing**			
	RD1-TRA	3'	TCGTCGAGGTTCTGGGTTCTGGATG
	RD1-TRB	3'	TCGTCGGGTGGAGTCACATTTCTCAG
	RD2	5'	GTGACTGGAGTTCAGACGTGTGCTCTTCCGATC
