# Supplementary material for: Molecular constraints on CDR3 for thymic selection of MHC-restricted TCRs from a random pre-selection repertoire
Source: Nat Commun. 2019 Mar 4;10:1019. doi: 10.1038/s41467-019-08906-7 (PMC6399321; doi:10.1038/s41467-019-08906-7)
Supplement: Supplementary file 3 — Reporting Summary [file 41467_2019_8906_MOESM3_ESM.pdf]

nature research

Corresponding author(s): Peter D. Sun
Last updated by author(s): Dec 20, 2018

Reporting Summary

Nature Research wishes to improve the reproducibility of the work that we publish. This form provides structure for consistency and transparency in reporting. For further information on Nature Research policies, see Authors & Referees and the Editorial Policy Checklist.

Statistics

For all statistical analyses, confirm that the following items are present in the figure legend, table legend, main text, or Methods section.

n/a	Confirmed	
☐	☒	The exact sample size (n) for each experimental group/condition, given as a discrete number and unit of measurement
☐	☒	A statement on whether measurements were taken from distinct samples or whether the same sample was measured repeatedly
☐	☒	The statistical test(s) used AND whether they are one- or two-sided<br>*Only common tests should be described solely by name; describe more complex techniques in the Methods section.*
☒	☐	A description of all covariates tested
☒	☐	A description of any assumptions or corrections, such as tests of normality and adjustment for multiple comparisons
☐	☒	A full description of the statistical parameters including central tendency (e.g. means) or other basic estimates (e.g. regression coefficient) AND variation (e.g. standard deviation) or associated estimates of uncertainty (e.g. confidence intervals)
☐	☒	For null hypothesis testing, the test statistic (e.g. F, t, r) with confidence intervals, effect sizes, degrees of freedom and P value noted<br>*Give P values as exact values whenever suitable.*
☒	☐	For Bayesian analysis, information on the choice of priors and Markov chain Monte Carlo settings
☒	☐	For hierarchical and complex designs, identification of the appropriate level for tests and full reporting of outcomes
☐	☒	Estimates of effect sizes (e.g. Cohen's d, Pearson's r), indicating how they were calculated

Our web collection on statistics for biologists contains articles on many of the points above.

Software and code

Policy information about availability of computer code

Data collection	All sequence data were collected by Adaptive Biotechnology, Irepertoire as indicated in Supplemental Table 1 & 2.
Data analysis	Preliminary data analysis was performed using public available software from Adaptive Biotechnology and Irepertoire.

For manuscripts utilizing custom algorithms or software that are central to the research but not yet described in published literature, software must be made available to editors/reviewers. We strongly encourage code deposition in a community repository (e.g. GitHub). See the Nature Research guidelines for submitting code & software for further information.

Data

Policy information about availability of data

All manuscripts must include a data availability statement. This statement should provide the following information, where applicable:

- Accession codes, unique identifiers, or web links for publicly available datasets
- A list of figures that have associated raw data
- A description of any restrictions on data availability

All data generated or analyzed during this study are included in this published article and its supplementary information files. They can be made available upon requests.

Field-specific reporting

Please select the one below that is the best fit for your research. If you are not sure, read the appropriate sections before making your selection.

☒ Life sciences ☐ Behavioural & social sciences ☐ Ecological, evolutionary & environmental sciences

Life sciences study design

All studies must disclose on these points even when the disclosure is negative.

Sample size	Sample size are standard practice in the field. A minimum of duplicate samples were provided whenever possible. Sample size varies between 2-9.
Data exclusions	No data are excluded.
Replication	All attempts to replicate data were successful.
Randomization	Not relevant
Blinding	Not relevant

Reporting for specific materials, systems and methods

We require information from authors about some types of materials, experimental systems and methods used in many studies. Here, indicate whether each material, system or method listed is relevant to your study. If you are not sure if a list item applies to your research, read the appropriate section before selecting a response.

Materials & experimental systems

n/a	Involved in the study	
☐	☒	Antibodies
☒	☐	Eukaryotic cell lines
☒	☐	Palaeontology
☐	☒	Animals and other organisms
☒	☐	Human research participants
☒	☐	Clinical data

Methods

n/a	Involved in the study	
☒	☐	ChIP-seq
☐	☒	Flow cytometry
☒	☐	MRI-based neuroimaging

Antibodies

Antibodies used	All FACS sorting antibodies were commercial and listed in the reagent section of the manuscript.
Validation	Validation of all commercial antibodies used here are listed on vendor's website.

Animals and other organisms

Policy information about studies involving animals; ARRIVE guidelines recommended for reporting animal research

Laboratory animals	Strains of mice are C57BL/6, BALB/c, B10.BR, QuadKO, QuadKO/Bcl2_tg, B6/Bcl2_tg. both sex animals were used with no preference.
Wild animals	no wild animals are used in the study.
Field-collected samples	Not relevant
Ethics oversight	all experiments involving animals were approved by NIH animal caring and use committee.

Note that full information on the approval of the study protocol must also be provided in the manuscript.

Flow Cytometry

Plots

Confirm that:

☒ The axis labels state the marker and fluorochrome used (e.g. CD4-FITC).

☒ The axis scales are clearly visible. Include numbers along axes only for bottom left plot of group (a 'group' is an analysis of identical markers).

☐ All plots are contour plots with outliers or pseudocolor plots.

☒ A numerical value for number of cells or percentage (with statistics) is provided.

Methodology

Sample preparation	T cells were isolated from either thymus or lymph nodes of various mice.
Instrument	BD FACSAria III is used to sort T cells
Software	FlowJo is used for all FACS data analyses
Cell population abundance	all abundance of post sorting cells are greater than 95% and indicated in Supplementary Figure S8.
Gating strategy	Gating strategy is described in the experimental method.

☒ Tick this box to confirm that a figure exemplifying the gating strategy is provided in the Supplementary Information.
