# Supplementary material for: Molecular constraints on CDR3 for thymic selection of MHC-restricted TCRs from a random pre-selection repertoire
Source: Nat Commun. 2019 Mar 4;10:1019. doi: 10.1038/s41467-019-08906-7 (PMC6399321; doi:10.1038/s41467-019-08906-7)
Supplement: Supplementary file 2 — Peer Review File [file 41467_2019_8906_MOESM2_ESM.pdf]

Reviewers' comments:

Reviewer #1 (Repertoire analysis, systems biology)(Remarks to the Author):

The authors present an interesting analysis of the pre- and post (thyme) selection TCR repertoires. Their data show that MHC restriction impacts (shortens) CDR3 length and severely reduces the usage of Cys and impacts aa usage. The overall conclusions are interesting and relevant to a broader audience. Having said that while the MS is appropriate for NC it requires major revision to address a number of technical issues.

Major:
1) The authors used the Adaptive TCR analysis pipeline for some mice and the iRepertoire for others. This is problematic because both the amplification protocols and sequence analyses are different and have different biases. Has there been an effort to compare TCR sequencing of T cells from the same animal by the two vendors?
2) Fig 1: The authors compared the top 100, 200 etc sequences based on number of identical reads. What is the rationale for binning based on number of reads?
3) Fig 1: Are the samples from total T cells or from naive cells? If not then presumably the high frequency TCRs must be from activated T cells!
4) The number of reads is heavily influenced by amplification biases and this is part of the reason why generally (unless one uses UMIs).
5) The authors do not explain how they analyze their data. I presume that the common sequences were determined after clustering in which case the clustering criteria need to be presented and also some analysis on whether the data is sensitive to changing the clustering criterion. Clustering is required to account for sequencing error!
6) I was surprised to see that the authors did not compare germline V usage. This kind of comparison can be quite informative and should be presented.
7) An analysis of public sequences among two or more animals would be helpful. In other studies public sequences are usually those that are detected in 3 or more subjects.
8) The difference in Trp usage between MHCr and MHCi in TCRb is quite pronounced and should be discussed.
9) It is very important to present the FACS gates for DN cells etc
10) Fig 5 does not add much to the analysis and should be moved to the SI. Same is true for Fig 9. In fact the message could be easily conveyed by a 5-6 total main Figure paper.

Minor:
1) Fig 1f and g are more important that panels 1a-e. I suggest reversing the order.
2) The authors may wish to discuss why Cys and Trp usage in MHCr and MHCi seems to vary by aa length. Any evidence for preferential J segment usage (although that may not be relevant for the composition of the F/G loop)

Reviewer #2 (Thymic selection, repertoire analysis)(Remarks to the Author):

The ability of the thymus to select appropriate TCR specificities expressed by thymocytes represents a key step in the formation and function of the immune system. Although thymic selection as a unique

thymic function has been examined in detail for many years, the processes remain obscure and enigmatic. Here, Lu et al examine TCR repertoire and thymic selection of MHC restricted and MHC independent TCR at multiple stages of thymus development. They show that clear differences exist in the selection mechanisms of these two distinct TCR repertoires. Their study shows that the thymus is able to support distinct positive selection processes that depend upon the specificity nature of the TCR. Overall, the study is very well performed, contains clearly thought out experiments, and the data is important in our understanding of how thymic selection mechanisms take place. I have several minor comments:

1. It is not fully clear why the authors, when examining the diversity of the pre-selection repertoire, use both CD69- DN and CD69-DP thymocytes. The latter are tought of as the typical substrate of thymic selection. With the former, can the authors rule out these cells are in fact pre-selection, and not mature DNabTCR+ T-cells?
2. As the positive selection window in vivo is constrained by the short (3-4d) lifetime of DP thymocytes, can the authors speculate on whether MHCi and MHCr TCRs are equally affected by this timeframe?
3. The peripheral T-cells used for analysis will contain both conventional and Foxp3+ Treg. Given the evidence for distinct intrathymic selection events for these 2 cell types, can the authors speculate on how their repertoires may be distributed within the pre-selection repertoire?

We thank both reviewers for their in-depth review of our manuscript and for their supportive comments. We have carried out additional analyses regarding to the CDR3 amino acid usages in public sequences and to the usage disparity of Trp and Tyr between MHCr and MHCi TCRs as suggested by the reviewers. We revised the manuscript according to their thoughtful comments. We think we have addressed all of their comments and the revised manuscript is significantly improved.

The following details point-by-point response to reviewer's comments with their comments highlighted in italic.

Reviewer 1

Reviewer #1 (Repertoire analysis, systems biology)(Remarks to the Author):
The authors present an interesting analysis of the pre- and post (thyme) selection TCR repertoires. Their data show that MHC restriction impacts (shortens) CDR3 length and severely reduces the usage of Cys and impacts aa usage. The overall conclusions are interesting and relevant to a broader audience. Having said that while the MS is appropriate for NC it requires major revision to address a number of technical issues.
Major:
1) The authors used the Adaptive TCR analysis pipeline for some mice and the iRepertoire for others. This is problematic because both the amplification protocols and sequence analyses are different and have different biases. Has there been an effort to compare TCR sequencing of T cells from the same animal by the two vendors?

Response: The reviewer indeed raised an important technical issue that commonly present in any sequencing related work. We were aware of this issue and indeed performed the cross validation exactly as the reviewer suggested including splitting samples between two vendors (original Supplemental Figure S8, renamed as Figure S9 in the revised manuscript). This cross validation sequencing, however, was not clearly indicated in the supplemental Table and as a result may not be apparent to the reviewer. We thank the reviewer for bringing up the point and will highlight the cross validation effort of our manuscript and our revision to further clarify the issue below. Different sequencing facilities often produce somewhat different PCR amplification results despite their effort to optimize PCR amplification and reduce primer-biases. These primer-bias associated systematic error can sometimes produce quite significant differences and compound our interpretation of the results, especially in regard to gene enrichment quantification or the frequency of sequences. In fact, that is one of the reason we use three different facilities, Adaptive Biotechnology, Irepertoire Inc, and National Institute of Aging (NIA) so that we can cross validate our results. While most of our TCR-beta repertoire sequencing were performed by Adaptive Biotechnology, we sequenced 7 duplicating TCR-beta repertoires through Irepertoire to serve as cross validation. They include three mature B6 repertoires (3mice), two mature BALB/c, two pre-selection DP from $MHC^{-/-}$ animals. They are listed in the original Supplementary Table 1. Moreover, the B6 and BALB/c duplicates were performed using T cells isolated from the exact same animals and split into two samples for Adaptive and Irepertoire. To better reflect the origin of these samples, we revised the sample ID to be the same as mouse ID and added a footnote to indicate the identical samples in Supplementary Table

1. We found from these cross validation sequencing are the following. While the primer-biases exist and vary among different sequencing facilities, they affect mostly V-gene frequencies as the primers are designed to be unique for the V-genes. The biases do not significantly affect CDR3 length and Cys content. A comparison of CRD3 length and Cys content from these duplicating sequencing data by the three sequencing facilities showed agreement in both CDR3 length and Cys content among these sequencing facilities (see supplemental Figure S8 in the original submission) (renamed as Figure S9 in the revised manuscript). In addition, we analyzed Cys contents in TCR-beta D-gene region to further reduce the influence of V-gene associated primer biases to our conclusion. Even for the V-gene usage, which is most affected by the primer biases, we found the systematic biases are minimized when the comparing repertoires are obtained from the same facility. That is these systematic biases tend to cancel out if compared within a facility.

2) Fig 1: The authors compared the top 100, 200 etc sequences based on number of identical reads. What is the rationale for binning based on number of reads?

Response: The reason for binning based on their frequency is to better represent in vivo circulating T cell population. As high frequency TCRs count for more circulating T cells with fewer sequences than low frequency TCRs, an equal unique sequence bin disregard of their frequencies would concentrate larger percentage of productive T cells in small number of bins at the high frequency end of the spectrum. For example, if we compare the TCR-beta sequences between two Quad deficient animals using equal unique sequence number bin. A 10 frequency intervals for Quad_1, that has ~50,000 unique sequences, would result in binning every 5000 sequences. Ideally, each frequency bin with 10% unique sequences should represent 10% of productive T cell population. Due to the uneven frequency distribution, the first bin (highest frequency) of the top 5000 sequences counts for ~50% of productive frequency. The last bin (lowest frequency) counts for less than 5% of the productive frequency. On the other hand, a reads or frequency-based binning results in more evenly distribution of the productive sequences. In the case of the Quad repertoire, the top 100 sequences count for 19.9% of productive sequences and the last bins count for ~20% (between 10,000 and 40,000), ~20% (between 2000 and 10000) productive sequences. Another reason is the number of unique sequences varies in the equal number binning with sequencing depth of each repertoire. Top 10% sequences may mean 5000 or 20000 sequences, making it difficult to compare between animals. A fixed number bin based on reads avoid this problem, especially sequencing depth generally affect the completion of low frequency sequence numbers more than high frequency ones.

3) Fig 1: Are the samples from total T cells or from naive cells? If not then presumably the high frequency TCRs must be from activated T cells!

Response: We thank the reviewer for asking this important question as the TCR repertoires from activated T cells are likely different from those of Naïve T cells. Our focus in this manuscript is on naïve not activated TCR repertoires. Specifically, we sorted naïve T cells based on $\gamma\delta TCR^-/TCR\beta^+$, and $CD44^-$ /$CD62L^+$ gates (see response to comment 9 for further details).

4) The number of reads is heavily influenced by amplification biases and this is part of the reason why generally (unless one uses UMIs).

Response: As the comment was an incomplete sentence, we think the reviewer is raising a related issue to the above comment 1. That is one way to offset the amplification biases is to use UMI-based method, which tags individual RNA prior to PCR amplification. While most of our TCRb sequencings were performed by Adaptive Biotech, we indeed cross validated our results using UMI method (see Supplementary Figure S8). The results from both companies are consistent with those by UMI method from the NIA facility. Thus, while PCR biases without UMI certainly present, they do not significantly affect our results.

5) The authors do not explain how they analyze their data. I presume that the common sequences were determined after clustering in which case the clustering criteria need to be presented and also some analysis on whether the data is sensitive to changing the clustering criterion. Clustering is required to account for sequencing error!

Response: Clustering analysis is used in various sequencing facilities to determine reads associated with individual sequences. It is part of sequencing bioinformatics performed prior to our analysis. Here, common sequences mean identical TCR amino acids between two sequences present in two repertoires. We account for errors present in our data through statistical approach of sequencing at least three animals from each type of repertoire. The consistency in our results is supported by statistical analysis.

6) I was surprised to see that the authors did not compare germline V usage. This kind of comparison can be quite informative and should be presented.

Response: We did compare both germline Va-gene and Vb-gene usages by pie-chart and by Pearson correlation coefficients (Original Figure 7 and 8, revised Figure 6 and 7). The results are described in a section titled:'Effect of thymic selection on V- and J-gene usages'. While variation in V-gene usages were observed in various animals, a comparison by Pearson correlation coefficient showed no significant preferences in Vβ and Vα usages distinguish MHCr from MHCi repertoires. For example, the Vβ and Vα usages of B6 resemble more to those of Q and QB than to those of B10.BR and BALB/c (Fig 7a-b), suggesting that the majority of V-gene frequencies were not affected by thymic selection.

7) An analysis of public sequences among two or more animals would be helpful. In other studies public sequences are usually those that are detected in 3 or more subjects.

Response: Following the reviewer's comment, we have further analyzed amino acid usage in public sequences that are defined as identical among three animals in addition to common sequences between two animals. Specifically, we identified all public TCR-beta sequences associated with each mice strain as well as from the pre-selection DNDP sequences, and analyzed their CDR3 FG-loop amino acid usage. The results are quite consistent with those from

two animals. Compared to the pre-selection public repertoire, Cys is absent in all MHC-restricted public repertoires and the usage of positively charged amino acids are significantly reduced. We have revised the manuscript to replace the common sequence analysis (original Figure 8c) with a new public sequence analysis (revised Figure 7c-d) as the public sequence analysis is more stringent than that of common sequence.

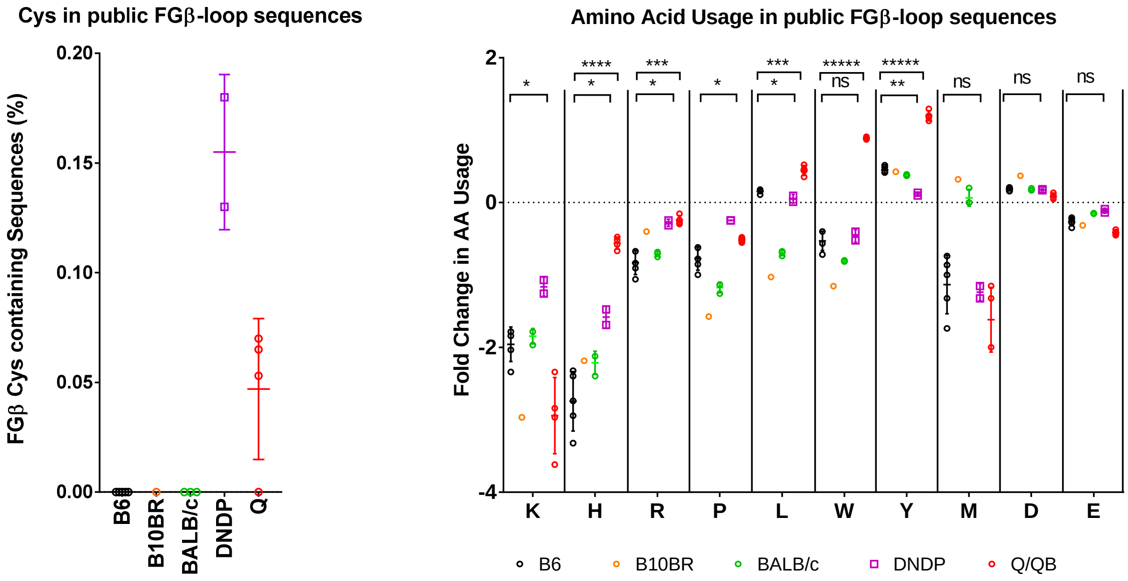

Figure 7c-d) Cys and other amino acid usages of CDR3β FG-loop among public sequences from MHCr or MHCi repertoires. The fold change is calculated with respect to those present in B6_DN repertoire. The public sequences of Q and QB are analyzed together.

8) The difference in Trp usage between MHCr and MHCi in TCRb is quite pronounced and should be discussed.

Response: We agree with the reviewer. Apart from the differences in CDR3 length and the FG-loop Cys frequencies, the usage of Trp differed remarkably in MHCr and MHCi CDR3 sequences. In fact, the frequencies of Tyr is also significantly higher in MHCi than MHCr TCR-beta. The differences are primarily due to MHCi animals promote the usage of both Trp and Tyr to higher frequencies than their pre-selection frequencies (Supplementary Figure S5n). In contrast, their frequencies in MHCr animals are similar to their pre-selection frequencies, which are also close to their respective amino acid occurrence frequencies in mammalian proteins (Gaur, IIOAB J. 5: 6-11, 2014). Like Trp and Tyr frequencies in MHCi CDR3, their frequencies in antibody CDR3H are also elevated compared to their respective amino acid frequencies in proteins, further demonstrate that TCR in the Q and QB animals are not MHC-restricted and they interact with native ligands like antibodies. The frequent occurrence of these two amino acids in antibody CDR3 presumably reflect their ability to engage significant higher hydrophobic interactions and thereby enhance ligand binding affinity. That is they are high affinity enabling

amino acids. We have revised the manuscript to expand the discussion on this point as suggested by the reviewer.

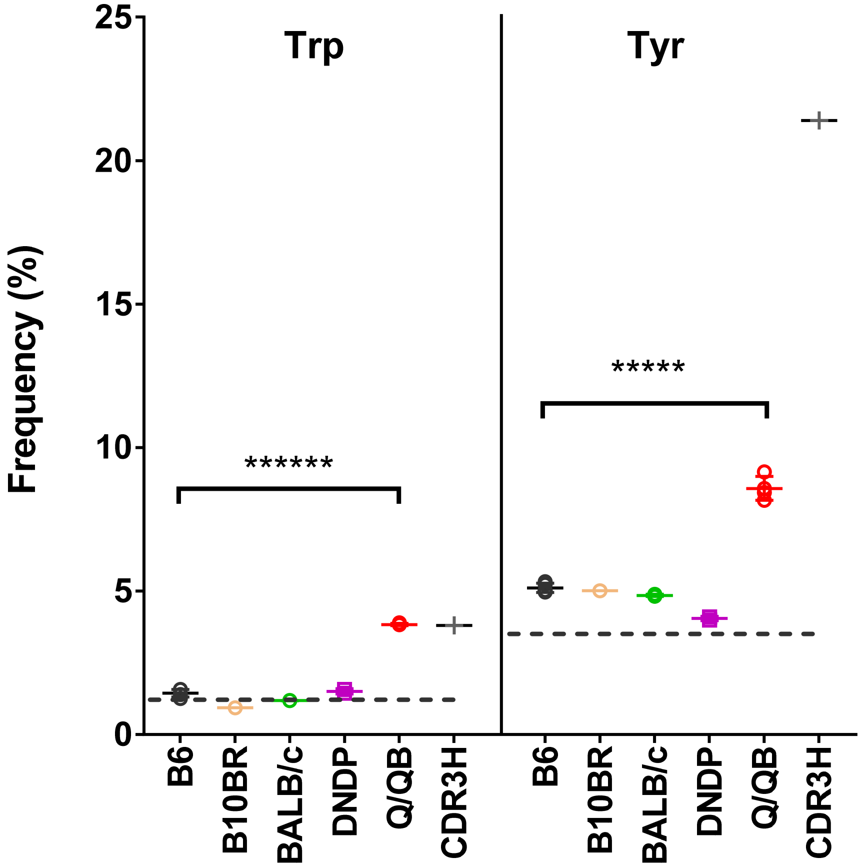

Supplementary Figure S5n. Observed frequencies of Trp and Tyr in the public CDR3β FG-loop of MHCr, MHCi as well as pre-selection repertoires. The TCRβ sequences are public sequences of each strain, namely common sequences observed in at least three animals of their individual strains. As a comparison, their respected frequencies in mammalian proteins are indicated by dashed lines and their frequency in antibody CDR3H are indicated by (+).

9) It is very important to present the FACS gates for DN cells etc

Response: As suggested by the Reviewer, we now include a new Supplementary Figure (Supplementary Figure S8) that defines the FACS sorting gates used to isolate the various T cell populations whose TCR repertoires we analyze in our study. Specifically, DN thymocytes were first enriched by magnetic depletion of NK1.1$^+$,B220$^+$, γδTCR$^+$, CD4$^+$, CD8β$^+$ and TCRβ$^+$ populations, and then sorted as CD4$^-$,CD8$^-$ and CD69$^-$ unsignaled DN thymocytes. The pre-selection unsignaled DP thymocytes were sorted as CD4$^+$, CD8α$^+$, and CD69$^-$ thymocytes from

B6 or MHC-KO animals. Naïve mature LN αβT cells were obtained by first magnetic depleting Ig^+ B cells, then FACS sorting based on $\gamma\delta TCR^-/TCR\beta^+$, and $CD44^-$ /$CD62L^+$ gates.

10) Fig 5 does not add much to the analysis and should be moved to the SI. Same is true for Fig 9. In fact the message could be easily conveyed by a 5-6 total main Figure paper.

Response: Figure 5a and 5b shows the frequencies of 20 amino acids in the FG-loop of CDR3β and CDR3α from individual MHCr, MHCi and pre-selection repertoires. Among them, several amino acids exhibited significant frequency differences between the pre-selection and mature repertoires as well as preferences associated with MHCr or MHCi repertoires. These amino acids, including Cys, charged and hydrophobic amino acids were further analyzed and their usages were presented as statistical means and standard variations in Figure 6. Even though Figure 5 and 6 describe the amino acid usage in CDR3 differently, we feel we can adequately describe the findings with Figure 5 moved to SI. We have thus moved original Figure 5a -b to SI as suggested by the reviewer and combined original Figure 5 and 6 as revised Figure 5 in the revised manuscript. Original Figure 9 describes the peripheral fitness of MHCr animals based on their frequency distribution. Up to this figure, the frequency of each sequence (reads) was used for binning the TCR sequences for overlap analysis. Here, we describe not only the frequency correlation between sequences from different repertoires, but also attempt to trace the "lineage" of mature sequences back to the pre-selection stage to examine their peripheral fitness. This figure does not overlap with the previous figures and makes an important scientific point regarding to influence of MHCr thymic selection to the peripheral fitness. We feel moving this figure to SI would undermine the illustration of the section. We thus decided to keep this figure as Figure 8 in revised manuscript.

Minor:
1) Fig 1f and g are more important that panels 1a-e. I suggest reversing the order.

Response: While we agree with the reviewer with the significance of panel 1f, we attempted to reverse the panel order as recommended. However, this created difficulty in the logic and continuity of describing these findings. Since both panels are part of the same figure, we decided not to reverse their order.

2) The authors may wish to discuss why Cys and Trp usage in MHCr and MHCi seems to vary by aa length. Any evidence for preferential J segment usage (although that may not be relevant for the composition of the F/G loop)

Response: We thank the reviewer for bringing up this excellent point. Indeed, the usage differences of Trp, Tyr, Cys, Pro, Arg between MHCr and MHCi repertoires exhibited CDR3β length dependence. In most cases, except Cys, their usage differences remained relative constant between 10-14 residues of CDR3β, but became less significant for CDR3 longer than 15 or shorter than 8 amino acids. These CDR3 length dependent amino acid usage variations are consistent with our current finding that thymic selection promotes 8-13 amino acid CDR3. Namely, the amino acid usage is compounded by MHC-imposed CDR3 length restriction.

Within the optimal CDR3 length, the amino acid usage difference reflect peptide-MHC binding preferences of TCR, hence the signature of MHC-restriction. Longer or shorter CDR3 are less optimal for MHC-binding, and progressively lost the signature of MHC-restriction. The revised manuscript included this brief paragraph in the discussion section.

The J-segment contributes to the C-terminal end of the FG-loop and thus its usage may differ in MHCr and MHCi repertoires. We presented both Jβ- and Jα-segment usage in various MHCr and MHCi TCR repertoires in pie charts (revised Figure 6 panel b and d). Like V-gene usages, variations in J-gene usages are observed but we did not find significant usage preferences associated with MHCr versus MHCi repertoires. To further illustrate the J-gene usages, we revised the manuscript to include the Pearson correlation coefficient comparisons showing individual Jβ-gene frequency in various MHCr, MHCi and pre-selection repertoires (Supplementary Figure S6a). Overall, Jβ-segment usage is very similar among all animals with their Pearson correlation coefficients varying between 0.7 and 1. Their usages are less variant than the V-gene usages. Even so, the repertoires from the same strain of animals consistently showed better Jβ-gene usage correlations. This internal consistency is also observed among all pre-selection repertoires. While J-gene usage did not exhibit significant preference between MHCr and MHCi TCRs, their usages appear less correlated between the pre-selection and mature repertoires. It is not clear why the pre-selection repertoires showed slight different J-gene usages than both MHCr and MHCi mature repertoires. It is clearly not dependent on MHC but is consistent with a greater sequence diversity present in the pre-selection repertoires than either mature ones.

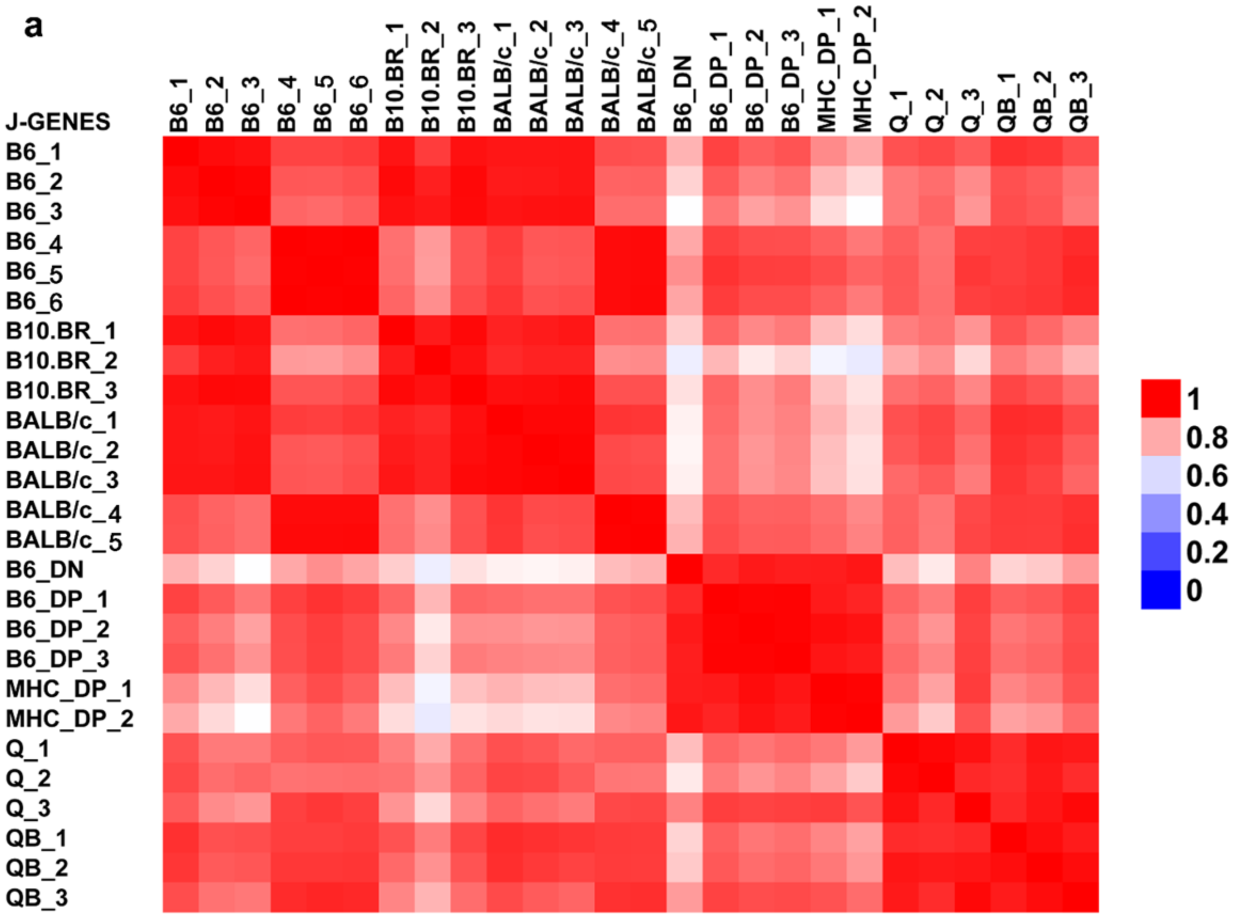

Supplementary Figure S6a. Pearson correlation coefficient calculation of Jβ-segment frequencies between various MHCr, MHCi and pre-selection repertoires.

Reviewer 2

Reviewer #2 (Thymic selection, repertoire analysis)(Remarks to the Author):
The ability of the thymus to select appropriate TCR specificities expressed by thymocytes represents a key step in the formation and function of the immune system. Although thymic selection as a unique thymic function has been examined in detail for many years, the processes remain obscure and enigmatic. Here, Lu et al examine TCR repertoire and thymic selection of MHC restricted and MHC independent TCR at multiple stages of thymus development. They show that clear differences exist in the selection mechanisms of these two distinct TCR repertoires. Their study shows that the thymus is able to support distinct positive selection processes that depend upon the specificity nature of the TCR. Overall, the study is very well performed, contains clearly thought out experiments, and the data is important in our understanding of how thymic selection mechanisms take place. I have several minor comments:
1. It is not fully clear why the authors, when examining the diversity of the pre-selection

repertoire, use both CD69- DN and CD69-DP thymocytes. The latter are thought of as the typical substrate of thymic selection. With the former, can the authors rule out these cells are in fact pre-selection, and not mature DNabTCR+ T-cells?

Response: While we examined the diversity of both DN and DP thymocytes, we only presented the diversity of DP in Figure 3a,b. We agree with the reviewer that since thymic selection takes place at DP stage and affected the development of DP thymocytes to mature T cells, the most meaningful sequence diversity comparison is thus between DP and mature repertoires, which is what we presented. DN population, however, offers another independent assessment to the pre-selection diversity of TCRβ and the result is consistent with that from DP thymocytes. Since DN data was not presented in Figure 3a,b, we revised the manuscript to clearly state the diversity of DP thymocytes.
These pre-selection DN cells are not mature DN αβTCR+ T cells as both αβTCR+ and γδTCR+ cells were depleted before sorting of the DN thymocytes. We added Supplementary Figure S8 to illustrate the selection and sorting schemes of various T cells. Please also see response 9 to reviewer 1.

2. As the positive selection window in vivo is constrained by the short (3-4d) lifetime of DP thymocytes, can the authors speculate on whether MHCi and MHCr TCRs are equally affected by this timeframe?

Response: We have no evidence to suggest MHCr and MHCi TCRs are differentially affected by the thymic selection kinetics. Our previous publication showed that MHCi TCRs are developed in the absence of MHC and their co-receptors as a result of binding to high affinity native ligands, such as CD155. It is likely the affinity threshold for MHCi TCR is much higher than that for MHCr TCR, which uses the co-receptors to lower their signaling threshold. The change in signaling threshold is expectedly to reduce the number and antigen-specific population of mature MHCi lymphocytes but may not alter their development kinetics since both MHCr and MHCi T cells signal through Lck and share the same intracellular signaling network.

3. The peripheral T-cells used for analysis will contain both conventional and Foxp3+ Treg. Given the evidence for distinct intrathymic selection events for these 2 cell types, can the authors speculate on how their repertoires may be distributed within the pre-selection repertoire?

Response: The reviewer raised a very interesting question. That is can one distinguish the conventional pre-selection from Treg pre-selection repertoires in the overall pre-selection repertoires. To do this, we need to know if conventional and Treg repertoires have distinct repertoire features, including amino acid usages, etc. Although such work is beyond the scope of this manuscript, we have revised the manuscript to provide further discussion: “It is worth noting that the CDR3 amino acid usages not only distinguish between MHCr and MHCi repertoires, preference also observed between CD4 and CD8 T cells within the MHCr repertoire (Supplementary Figure S5m). It is conceivable that other mature T cell populations, such as

conventional and Foxp3+ Treg T cells, may also exhibit distinct repertoire properties that allow the population be identified at sequence level."

REVIEWERS' COMMENTS:

Reviewer #1 (Remarks to the Author):

I am pleased with the authors responses to the issues I raised in the first round of review. There is one outstanding issue to be addressed, which however in my view does not necessitate a second re-review: Regarding #5 the authors respond that clustering was performed by the different repertoire sequencing facilities. However the different informatics analyses likely involved different criteria for clustering and these may affect the analysis. Hence the authors should attempt to provide information (which the sequencing facilities must have available) on how clustering was performed. Absent that I suggest that the authors perform a quick re-analysis of the CDR3 sequencing data with a clustering threshold of 90% or 95%. This may be conservative since the authors state that clustering may already have been done (however we do not know how) but at least a second clustering might yield a baseline (and very conservative) estimate diversity etc. Just for peace of mind...

Reviewer #2 (Remarks to the Author):

The authors have considered my comments appropriately

Point-to-point Response to the Reviewer's Comments

We thank both reviewers for their supportive comments. While reviewer 2 is satisfied with our revision, reviewer 1 suggested again for us to re-analyze the sequences using different clustering criteria to address if the conclusions are affected by biases in various sequencing facilities. We had length discussions with the technical experts at Adaptive Biotechnologies, Inc. The Adaptive experts did not think our conclusions would be affected by their sequence clustering criteria, and did not agree for carrying out additional cluster analysis on our sequences citing lack of proper bioinformatic tools. We subsequently performed a re-clustering analysis as suggested by the reviewer and found it did not affect our conclusions. Below lists our detailed response to comments from the reviewer with the comments highlighted in italic. The response to the editor's comments are addressed in the reply section of respective comment bubbles.

Reviewer 1.

I am pleased with the authors responses to the issues I raised in the first round of review. There is one outstanding issue to be addressed, which however in my view does not necessitate a second re-review: Regarding #5 the authors respond that clustering was performed by the different repertoire sequencing facilities. However the different informatics analyses likely involved different criteria for clustering and these may affect the analysis. Hence the authors should attempt to provide information (which the sequencing facilities must have available) on how clustering was performed. Absent that I suggest that the authors perform a quick re-analysis of the CDR3 sequencing data with a clustering threshold of 90% or 95%. This may be conservative since the authors state that clustering may already have been done (however we do not know how) but at least a second clustering might yield a baseline (and very conservative) estimate diversity etc. Just for peace of mind...

Response: We agree with the reviewer that different sequencing facilities process their sequencing data differently. Previously, we showed in supplemental Figure 9 that different sequencing facilities yielded essentially the same conclusions regarding to both the presence of shorter CDR3 lengths and the clonal deletion of cysteines in MHC-restricted TCR repertoires. Thus, the clustering criteria used by different sequencing facilities do not affect our conclusion. We further performed re-analysis of MHCr B6, the pre-selection double positive DP and MHCi Q and QB sequences using a 90-95% clustering threshold, as suggested by the reviewer. The results show that TCRβ sequences obtained after the clustering analysis exhibit similar CDR3 length distributions (Figure R1 of the response) and Cysteine usage (Figure R2) as those in Figure 1, 2 and 4 of the manuscript. That is MHC-restricted B6 mice contain shorter CDR3 and favors 8-12 amino acid CDR3 compared to MHCi Q and QB. Cys is absent in the peptide-MHC binding region of B6 CDR3 but present in the pre-selection DP as well as MHCi sequences.

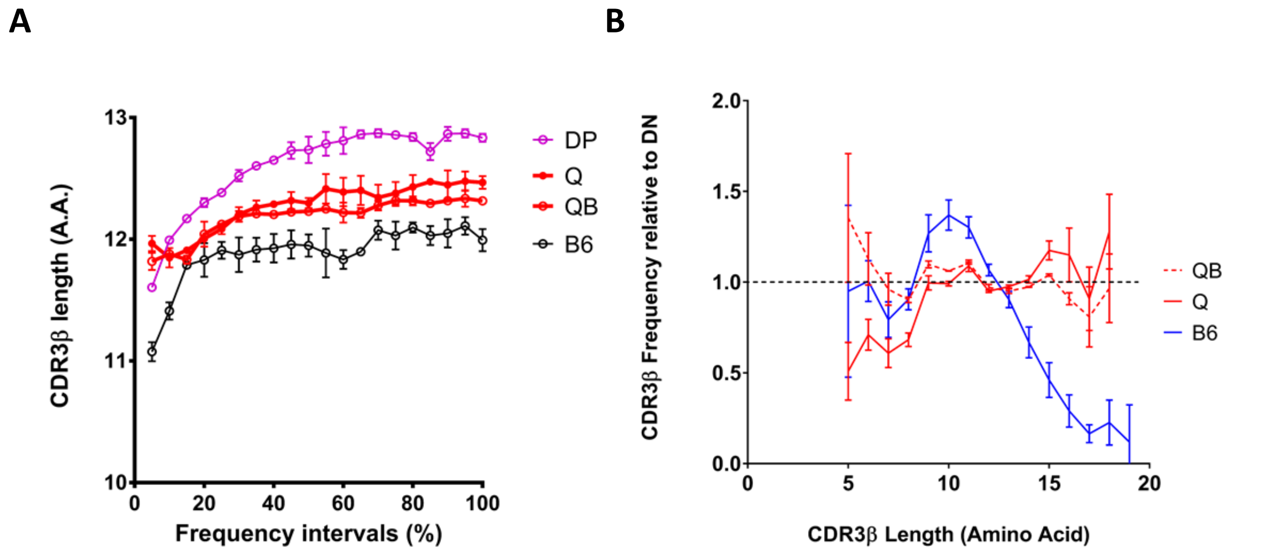

Figure R1. CDR3β length distribution analysis using sequences after clustering analysis. A) Frequency dependent CDR3β length distribution in MHC-restricted B6, pre-selection B6_DP, and MHC-independent Q and QB repertoires. B) Normalized CDR3β length distribution.

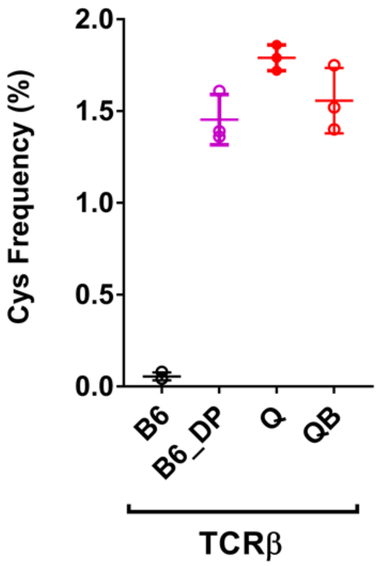

Figure R2. CDR3β FG-loop Cysteine usage in MHCr B6, pre-selection B6_DP and MHCi Q and QB repertoires. The frequencies are calculated after clustering analysis with 90-95% threshold.
